# *InstaMap*: instant-NGP for cryo-EM density maps

**DOI:** 10.1107/S2059798325002025

**Published:** 2025-03-26

**Authors:** Geoffrey Woollard, Wenda Zhou, Erik H. Thiede, Chen Lin, Nikolaus Grigorieff, Pilar Cossio, Khanh Dao Duc, Sonya M. Hanson

**Affiliations:** a Center for Computational Biology, Flatiron Institute, New York, NY10010, USA; b Center for Computational Mathematics, Flatiron Institute, New York, NY10010, USA; c Department of Computer Science, University of British Columbia, Vancouver, British Columbia, Canada; d Cornell University, Ithaca, New York, USA; e University of Massachusetts Chan Medical School, Worcester, Massachusetts, USA; f Department of Mathematics, University of British Columbia, Vancouver, British Columbia, Canada; National Center of Biotechnology, CSIC, Spain

**Keywords:** cryo-EM, density maps, heterogeneity, end-to-end gradient-based learning

## Abstract

Despite the parallels between problems in computer vision and cryo-electron microscopy (cryo-EM), many state-of-the-art approaches from computer vision have yet to be adapted for cryo-EM. Within the computer-vision research community, implicits such as neural radiance fields (NeRFs) have enabled the detailed reconstruction of 3D objects from few images at different camera-viewing angles. While other neural implicits, specifically density fields, have been used to map conformational heterogeneity from noisy cryo-EM projection images, most approaches represent volume with an implicit function in Fourier space, which has disadvantages compared with solving the problem in real space, complicating, for instance, masking, constraining physics or geometry, and assessing local resolution. In this work, we build on a recent development in neural implicits, a multi-resolution hash-encoding framework called instant-NGP, that we use to represent the scalar volume directly in real space and apply it to the cryo-EM density-map reconstruction problem (*InstaMap*). We demonstrate that for both synthetic and real data, *InstaMap* for homogeneous reconstruction achieves higher resolution at shorter training stages than five other real-spaced representations. We propose a solution to noise overfitting, demonstrate that *InstaMap* is both lightweight and fast to train, implement masking from a user-provided input mask and extend it to molecular-shape heterogeneity via bending space using a per-image vector field.

## Abstract 2

Cryo-EM density-map inference, with fixed pose and contrast transfer function, using a multi-resolution hash-encoding framework called instant-NGP, is described, together with its extension to heterogeneity inference by bending space with a per-image vector field.

## 1. Introduction

Cryogenic electron microscopy (cryo-EM) of biomolecules (for example proteins, nucleic acids and lipids) is a structural biology technique that images ultracooled specimens with phase-contrast transmission electron microscopy at magnifications on the subnanometre scale. In single-particle experiments, samples are purified and placed on a grid, and induced to form a thin film before being vitrified and imaged. The resulting images of millions of individual molecules are then recombined into a 3D structure or ensemble of structures (Brzezinski, 2017 ▸) and made publicly accessible to the community, enabling downstream applications (Jumper *et al.*, 2021 ▸; Varadi *et al.*, 2022 ▸; Kleywegt *et al.*, 2024 ▸; Turner *et al.*, 2024 ▸). In cryo-EM, as with many areas of structural biology (Corso *et al.*, 2024 ▸), computational biology (Wang *et al.*, 2024 ▸) and all of science (Lavin *et al.*, 2021 ▸), there is an increasing application of general tools from AI/ML and computer vision. Algorithms, programming languages and hardware exist in an asymmetric relationship (Hooker, 2021 ▸), and in diverse ways are combined with heuristics from bespoke analyses that were historically developed by domain scientists. In the case of cryo-EM, there is a strong tradition of signal processing approaches, numerical linear algebra, Bayesian inference, statistical inference and, more recently, differentiable programming/deep learning and simulation-based inference (Jensen, 2010 ▸; Singer & Sigworth, 2020 ▸; Donnat *et al.*, 2022 ▸; Dingeldein, Cossio *et al.*, 2024 ▸).

The field of computer vision has a long history of optimizing the estimation of 3D models from 2D images, as the applications in industry are vast, ranging from satellite image annotation (Blaschke, 2010 ▸) to self-driving cars (Bojarski *et al.*, 2016 ▸) to simulated video-game play (Eslami *et al.*, 2018 ▸). Recently, implicit neural representations in the form of neural scalar fields (Lu *et al.*, 2021 ▸) and neural radiance fields (NeRFs; Mildenhall *et al.*, 2022 ▸) have highly impacted computer vision by enabling the reconstruction of 3D objects from images at different camera-viewing angles. In a short time these methods and their variants have become commonplace in the analysis of natural images to generate a model of the 3D world that they represent. Even before its application to natural images, a NeRF-like architecture was used in a difficult scientific inference problem: to infer motions of 3D volumes of biomolecules from 2D cryogenic electron-microscopy (cryo-EM) data (Zhong, Bepler *et al.*, 2021 ▸). However, because cryo-EM images have different fundamental properties to natural images in terms (i) of high noise, (ii) of microscope effects and (iii) of the projective nature of the image-formation process, popular computer-vision approaches for natural images cannot be naïvely adopted out of the box. So how can we represent a biomolecule’s shape?

Like various other inverse imaging problems (Ongie *et al.*, 2020 ▸), cryo-EM has recently benefited from approaches that leverage neural representations of shape. In addition to *cryoDRGN* (Zhong, Bepler *et al.*, 2021 ▸), the cryo-EM literature now contains several uses of neural representations for the volume, for tasks of either homogeneous or heterogeneous reconstruction with or without knowledge of pose, and methods as recent as 2023 are reviewed in Donnat *et al.* (2022 ▸) and Toader, Sigworth *et al.* (2023 ▸). Various architectures have been used for volume representation in cryo-EM: (i) dense multilayer perceptrons (MLPs; Zhong, Bepler *et al.*, 2021 ▸; Rosenbaum *et al.*, 2021 ▸; Levy, Raghu *et al.*, 2022 ▸; Li *et al.*, 2024 ▸), (ii) the MLP-based *FourierNet* in Levy, Poitevan *et al.* (2022 ▸), which used sinusoidal activation functions (SIRENs) and element-wise exponentiation to cover the large dynamic range in Fourier space, (iii) a real-spaced SIREN approach (Herreros *et al.*, 2024 ▸), (iv) coordinate-based representations that map to deterministic density kernels (Chen & Ludtke, 2021 ▸; Chen *et al.*, 2023 ▸) and (v) 3D convolutional layers (Gupta *et al.*, 2020 ▸). Recent real-space approaches allow spatial locality and are efficiently composable with geometric operations on coordinates such as local coarse-graining, masking and regularization for smoothness and similarity to a reference atomic model (Chen & Ludtke, 2021 ▸; Chen *et al.*, 2023 ▸; Herreros *et al.*, 2024 ▸; Schwab *et al.*, 2024 ▸). Over six months after the initial submission of our white paper for this special issue, we became aware of concurrent work similar to our method (Qu *et al.*, 2025 ▸) that also employs instant-NGP to model cryo-EM density, including heterogeneity. While our approaches differ in some ways, the similarities are encouraging for the promise of applying instant-NGP to problems in cryo-EM.

Here, we also make a real-spaced choice for volume representation and employ a neural implicit function that outputs directly to real space. Our choice is motivated by the availability of a lightweight neural implicit function with multi-resolution hashing known as an instant neural graphics primitive: instant-NGP (see the tinycudann documentation for the PyTorch bindings; https://github.com/NVlabs/tiny-cuda-nn), which shows impressive performance for rapid training of 3D scenes from 2D natural images with known pose (Müller *et al.*, 2022 ▸). This lightweight architecture helps the real-space computation become tractable. In brief (see Fig. 3 of Müller *et al.*, 2022 ▸), for each query coordinate in 3D space, the surrounding voxels (in 3D space) are looked up at *L* resolution levels. For each of the corner indices touching the query coordinate, learnable feature vectors of length *F* are efficiently looked up through a hash table and linearly interpolated in 3D space. This intermediate encoding value of dimension length *LF* is passed to a trainable MLP, which here predicts the scalar density value at the coordinate. Gradient information is back-propagated through the MLP, including the concatenation of *L* feature vectors and the linear interpolation, to the trainable look-up feature vector of length *F*. To summarize, instant-NGP, as we have applied it, maps a spatial coordinate in 3D to a scalar density in 3D. As we will explain in Section 2.1 and Algorithm 1, other parts of the image-formation model of cryo-EM (the projection, application of microscope effects and modeling of heterogeneity) happen upstream or downstream of the instant-NGP architecture through a differentiable computation.
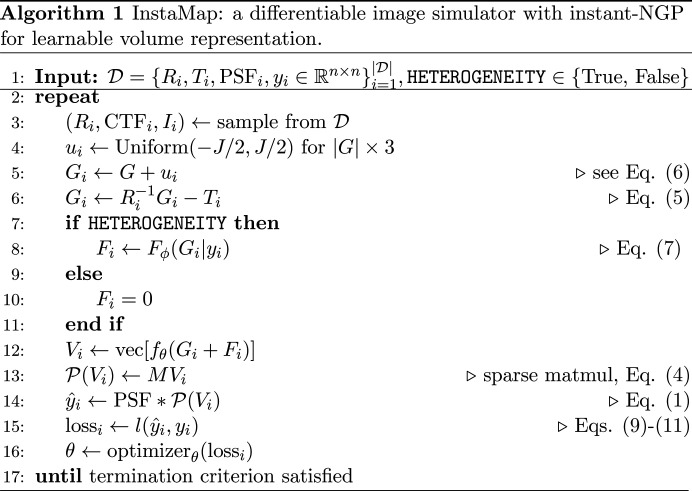


After presenting our implementation of instant-NGP for cryo-EM density maps, we perform experiments on both synthetic and real data, assuming that poses (rotation and translation) and imaging parameters (point-spread function/contrast transfer function) are known. We highlight advantages in the early training stages. We show results for several loss functions that are invariant to global multiplicative and additive scaling of the input particles, or that marginalize out different signal-to-noise ratios. We demonstrate masking of regions inside a pre-defined mask by subsetting coordinates. Finally, we extend *InstaMap* to the heterogeneous reconstruction problem in cryo-EM using per-image vector-field deformation and infer two-state discrete conformational heterogeneity.

## 2. Methods

**Figure 1:**
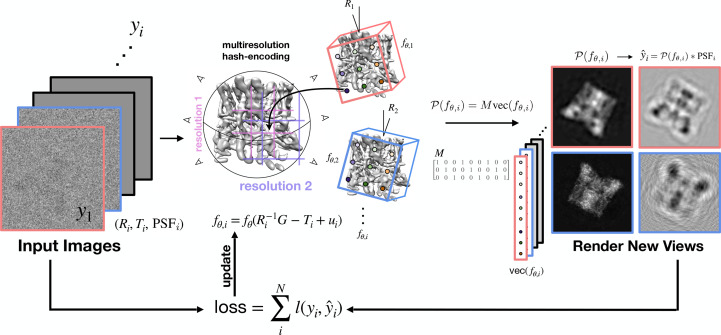
Overview of *InstaMap*. Cryo-EM images *y*_*i*_ (left) with annotated pose and imaging parameters (*R*_*i*_, *T*_*i*_, PSF_*i*_) are used for gradient-based learning. Instant-NGP is queried at the rotated, shifted and jittered grid. A pose-independent projection matrix maps the 3D grid indices to the 2D plane (middle). The electron-microscope effects are then applied to generate a noiseless projected image corresponding to the observation of the top view of the biomolecule (TRPV1 ion channel; right). The loss function is computed by summing the losses from the individual particles.

We perform reconstruction of the cryo-EM volume under the weak phase and projection approximations (Vulović *et al.*, 2014 ▸; Glaeser *et al.*, 2021 ▸), using instant-NGP as the learnable volume representation. We will now describe how we integrated the cryo-EM forward model with the learnable volume representation (see Fig. 1 ▸ and Algorithm 1).

### 2.1. Forward model of image formation

For a given scalar density representation 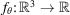, we apply the pose ∈ SE(3) [rotation *R* ∈ SO(3), translation 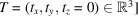 by rotating the grid points in the microscope’s frame, rather than rotating the specimen’s density in a fixed microscope frame. The rotated density is *f*_θ_(*x*, *y*, *z*) = *f*_θ_[*R*^−1^(*x*′, *y*′, *z*′)^*T*^ + (−*t*_*x*_, − *t*_*y*_, 0)^*T*^]. Fig. 1 ▸ shows examples of the rotated grids (blue and red boxes). Note how the (silver) 3D density remains in a fixed frame. After the rotation is applied, the observed image may be described as 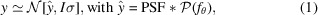
where 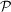 denotes orthogonal projection along the *z* axis, 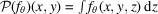 and 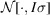 denote per-pixel independent and identically distributed (i.i.d.) Gaussian white noise with variance σ^2^, *I* is the identity matrix and (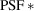) denotes the convolution with the point-spread function, which is a linear operator describing the application of the contrast transfer function (CTF) in Fourier space. Standard expressions for the CTF can be found in Wade (1992 ▸), with more contemporary notation in Rohou & Grigorieff (2015 ▸). We applied the parametric form of the CTF via element-wise multiplication in Fourier space, with further details given in Section A2. For the experiments in this work, these CTF parameters are supplied with the data and are not estimated by *InstaMap*.

### 2.2. Computational implementation of the forward model

We discretize the volume at grid coordinates 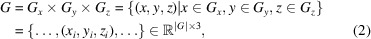
where the *x*, *y* spatial coordinates in *G*_*x*_ = *G*_*y*_ are on a regularly sampled line with the same diameter as the observable *y* (or within a user-specified mask, or inscribed within the sphere with the same diameter as *y*). Points *z* in the viewing direction are likewise regularly spaced, but their number (*G*_*z*_) is separately controlled. The exact layout of points in the axis-aligned grid is determined by two parameters per *xyz* spatial direction for a total of six: the number of points in each direction and the spacing between each grid point. For our experiments, we configured the number of points and the spacing in the imaging plane to correspond to one point per pixel in the observable *y*. The number of points in the viewing direction, |*G*_*z*_|, had 64 or 128 points, with spacing to cover the same extent as in the other directions. Thus, for an image size of 100 × 100 pixels, there would be 100 × 100 × 64 or 100 × 100 × 128 grid points (before masking).

As described in the forward model of image formation (Section 2.1), rather than rotating and translating a scalar density of a 3D volume, we query the implicit function *f*_θ_ at grid points corresponding to the desired pose. We project 3D density to 2D via sparse-matrix multiplication, 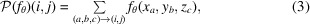
where (*a*, *b*, *c*) → (*i*, *j*) denotes the set of grid-coordinate indices that project to pixel (*i*, *j*). In other words, the projection is numerically implemented as a sparse-matrix multiplication of *f*_θ_, the scalar density at discrete points, and 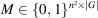, which has values of 1 where the mapping exists and 0 elsewhere. By querying the entire pose-specific grid *R*^−1^*G* − *T*, we can project through a sparse-matrix multiplication 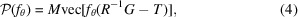
where vec denotes the ordering of density points corresponding to 3D locations as a (flattened) vector, with 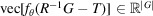. In general, the layout of points in *G* does not have to correspond to the same spacing as the image, or even be on a regular grid, which is why we use the notation |*G*| instead of *n*^3^ to denote the number of points. The mapping of 3D spatial points (*a*, *b*, *c*) → (*i*, *j*) is fixed under pose, and thus *M* is fixed and only needs to be computed once for the coordinates in *G*. In the case of dynamic masking, *G* changes to *G*′ and *M*′ contains a subset of columns corresponding to the grid points in *G*′.

Before querying *f*_θ_, we randomize the computation by jittering the sampling points in order to produce an estimate of the projection (more motivation for this can be found in Section 3.2). A jittered grid point at a specific pose is 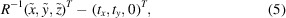

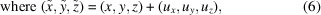
and where *u*_*x*_, *u*_*y*_, *u*_*z*_ are uniform random variables sampled i.i.d. from [−*J*/2, *J*/2], yielding one random variable per point coordinate for a total of |*G*| × 3 and scaled by 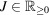. The posed and jittered points from equation (6) can reach all spatial points in a continuous manner: they are not on a fixed lattice and reach ‘in-between’ the original spacing of points in *G*. Thus, as a learnable continuous representation, with more particles the ‘resolution’ can exceed the limits from discretized representations, such as the Nyquist frequency in Fourier representations [see Li *et al.* (2023 ▸) for an example of super-resolution beyond Nyquist in photonics].

After querying *f*_θ_, jittering and projecting, we apply the CTF as a discrete convolution through an element-wise multiplication in Fourier space (see equation 14).

### 2.3. Instant-NGP multi-resolution hashed neural scalar field for 3D density

In general, in order to perform numerical optimization for 3D reconstruction, we must parametrize the density *f*_θ_ in some closed form amenable to computation (in both its forward pass and its gradient-based update). We note that the entire image-formation process (equation 1) is fully differentiable with respect to the samples *f*_θ_(*x*, *y*, *z*), and hence to have gradient updates to the global variable θ from the loss 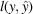, it suffices to obtain a differentiable parametrization of 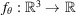. Consequently, we make use of a neural implicit representation of the cryo-EM map’s scalar density. We do not predict color and specularity, as they do not appear in the cryo-EM imaging process. Moreover, unlike most NeRF applications with natural images, our problem is free from observational differences in the aspect (gloss, reflectance, specularity) of the object from different views, because our goal in cryo-EM reconstruction is to recover a latent representation of the object that is independent from perspective.

As our specific choice of model, we apply a state-of-the-art technique issued from the computer-vision literature: instant-NGP (neural graphics primitives; Müller *et al.*, 2022 ▸). We perform experiments on other options for the scalar density representation, *f*_θ_, to verify that instant-NGP provides a favorable balance between the number of parameters required to represent the signal, the quality of the reconstruction, the computational power required to evaluate the function and the training time. For our instant-NGP multi-resolution hash encoder we used the tinycudann PyTorch bindings. Our setup is akin to that of the original instant-NGP publication (Müller *et al.*, 2022 ▸) and employs eight geometrically scaled levels. In configuring the encoder for various biomolecules of different box lengths (number of pixels), we tailored the maximum size in the instant-NGP multi-resolution hash encoder to vary, adapting to the unique requirements of each biomolecule. As for the architecture of the decoder, we followed a design similar to the original publication, utilizing two two-layer MLP decoders, each equipped with 64 (in some experiments 128) neurons and ReLU activations.

### 2.4. Heterogeneity

To incorporate heterogeneity into the *InstaMap* framework, we infer a per-image vector field, 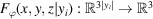, parametrized by a global variable φ, and then ‘bend’ the spatial grid coordinates by a per-image vector field, before querying the scalar density parametrized by instant-NGP. This was performed through a neural network (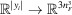) conditioned on each image that maps to a regular grid of shape (3, *n*_*F*_, *n*_*F*_, *n*_*F*_), which represents a discretized vector field *F* downsampled from the original image by the ratio *n*_*F*_/*n*. We linearly interpolate the vector field at the same pose-specific grid points as the scalar field (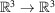). Since the output of the neural network layers is pose-invariant (*i.e.* it has a canonical reference frame like *f*_θ_), and the linear interpolation is performed at the pose-specific grid points, *G*_*i*_, jittered by uniform noise, *u*_*i*_ (see equation 6), the pose-specific vector field *F*_*i*_ is SE(3) equivariant. Here, we apply *F*_*i*_ to *G*_*i*_ via addition, 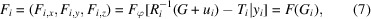

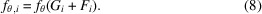


### 2.5. Loss functions

Within our *InstaMap* framework we implemented three loss functions that all arise from a Gaussian white-noise model. These loss functions are at one ‘end’ of our ‘end-to-end’ approach. In any one experiment we numerically auto-differentiate one of them with respect to the high-dimensional global variables θ, φ.

#### 2.5.1. Mean-squared error

The most basic loss function we use is the mean-squared error (MSE), arising from the generative model where the image pixels are i.i.d.: 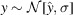. MSE is the negative log-likelihood of this model. The corresponding loss function is 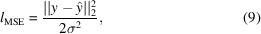
where σ is the standard deviation (assumed to be known) and 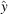 is the pixel image intensity from the forward model (equation 1) without noise.

#### 2.5.2. Cross-correlation

We also implemented a cross-correlation (CC) loss that is invariant to global multiplicative and additive scaling (proof in Appendix A4[App appa], equation 18). It is related to the cross-correlation between the observed and calculated forward model images via 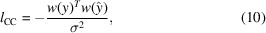
where 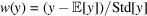 and where 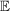 and Std are the expected value and standard deviation, respectively. Unless otherwise stated, we used *l*_CC_ in our experiments with σ = 3, which merely numerically scales the loss by a constant.

#### 2.5.3. BioEM

Finally, we implemented the Bayesian inference of electron microscopy (BioEM) loss function, given by 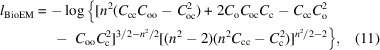
where *n*^2^ is the number of pixels, 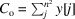, 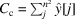, 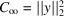, 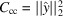, 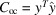, *j* indexes the pixels and o and c stand for observed and calculated, respectively. This loss does not involve an estimate of the noise level (σ in equations 9 and 10). Cossio & Hummer (2013 ▸) originally developed this loss for Bayesian inference of electron microscopy (BioEM) images by marginalizing over all global multiplicative and additive scalings with a uniform prior, and performing a saddle-point approximation to integrate out the noise parameter σ (equation 10 in their Supplementary Information). We experienced a numerical issue, because the original BioEM loss is minimized by minimizing the magnitude 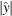. In our case this resulted in an instability due to finite precision, where 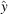 was driven to all zeros. We overcame this numerical issue by re-deriving the loss under a Gaussian prior. We explain this at length in Section A4.

### 2.6. Data sets

To show the efficacy of the *InstaMap* approach on homogeneous reconstruction, we analyze both synthetic and real cryo-EM data sets of two proteins: the TRPV1 ion channel and apoferritin. Synthetic data were simulated from the atomic model, where the density was approximated with a mixture of Gaussians placed at each atom coordinate, corresponding to the parametrization in Lobato & Van Dyck (2014 ▸), and added Gaussian white noise at fixed σ = 3, resulting in signal-to-noise (SNR) ratios of 0.050 ± 0.005 (Fig. 13). For the real experimental (empirical) cryo-EM data sets we used the publicly available TRPV1 (EMPIAR-10005) and apoferritin (EMPIAR-10421) data sets. The distribution of pose and microscope parameters of empirical data was used to generate synthetic data. For heterogeneous reconstruction we used a coarse-grained model of thyroglobulin with higher signal (σ = 0.1). The number of particles used in each experiment ranges from less than 1000 (in an experiment set up to promote overfitting; see Section 3.2) to as large as 80 000, and is mentioned in place. Further details are given in Section A5.

## 3. Experiments

### 3.1. *InstaMap* representations achieve higher resolution in early training

**Figure 2:**
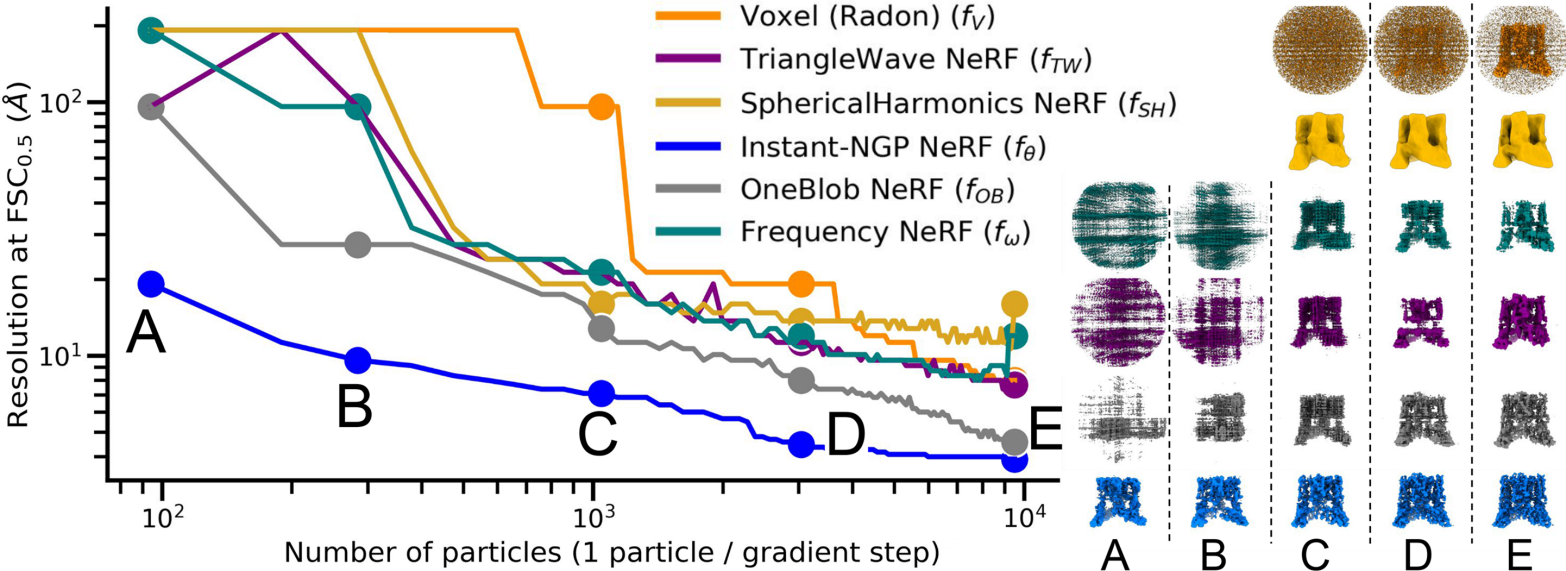
Instant-NGP trains fast. Resolution of the TRPV1 ion channel using instant-NGP versus various real-spaced representations for synthetic data (10 000 particles). Volume resolution, measured with the Fourier shell correlation (FSC), is shown as a function of the gradient-training steps for varous real-spaced volume representations. All representations have a similar number of training parameters (∼20 000), except for the voxel representation which has 4.1 million, ∼20× more. The FSC was calculated against the reference structure from which the synthetic data was generated, with a threshold value of 0.5 used to estimate the resolution. Example 3D volumes are shown at the indicated time points (A, B, C, D and E).

In order to highlight the benefit of instant-NGP’s multi-resolution hash compared with other parametrizations of the scalar field, we compared instant-NGP against five other volume representations using the same algorithmic framework and code base: *f*_θ_ denotes an instant-NGP with multi-resolution hash Grid encoding (Müller *et al.*, 2022 ▸), *f*_ω_ denotes a ‘frequency neural implicit’ (sinusoidal encoder and MLP decoder; Mildenhall *et al.*, 2020 ▸), *f*_TW_ denotes a TriangleWave-encoded (Müller *et al.*, 2021 ▸) neural implicit, *f*_OB_ denotes a OneBlob-encoded neural implicit (Müller *et al.*, 2019 ▸, 2020 ▸), *f*_SH_ denotes a spherical harmonics encoded neural implicit^1^ and finally *f*_V_ denotes a real-space voxel intensity, interpolated at any pose-specific grid point through torch.nn.functional.grid_sample(..., mode=‘bilinear’, padding_mode=’zeros’). Each of these functions performs the same mapping from coordinates to real-space density. Fig. 2 ▸ compares the map resolution, measured with the Fourier shell correlation (FSC), over the course of training for the synthetic TRPV1 data. The FSC was calculated between the 3D rendering from each of *f*_V_, *f*_TW_, *f*_SH_, *f*_θ_, *f*_OB_ and *f*_ω_ compared with the ground-truth volume from which we generated the synthetic data, and the 0.5 threshold was used was used, which we denote by FSC_0.5_. Examples of the rendered densities are also shown for each method (blue, green and orange). Remarkably, instant-NGP renders volumes around ∼4 Å resolution after training on only a few hundred images (Fig. 2 ▸). This is in contrast to *f*_V_, *f*_TW_, *f*_SH_, *f*_OB_ and *f*_ω_, even though they have a similar number of trainable parameters of ∼200 000 or more (*f*_V_ has ∼4 000 000).

The noise for the instant-NGP is also unique: there is little ‘noise dust’ in the intermediate renderings, which is consistent with the multi-resolution hash of instant-NGP (A–E in Fig. 2 ▸). Overall, our results suggest that much less training time is needed, and that low-resolution features can be learned from a few hundred images (with known poses). We also quantify this trend for empirical data: Fig. 11 shows that *InstaMap* achieves better resolution than DFI, reaching ∼10 Å with ∼60 particles and ∼3 Å with 20 000 particles.

### 3.2. Jittering grid points reduces noise overfitting

**Figure 3:**
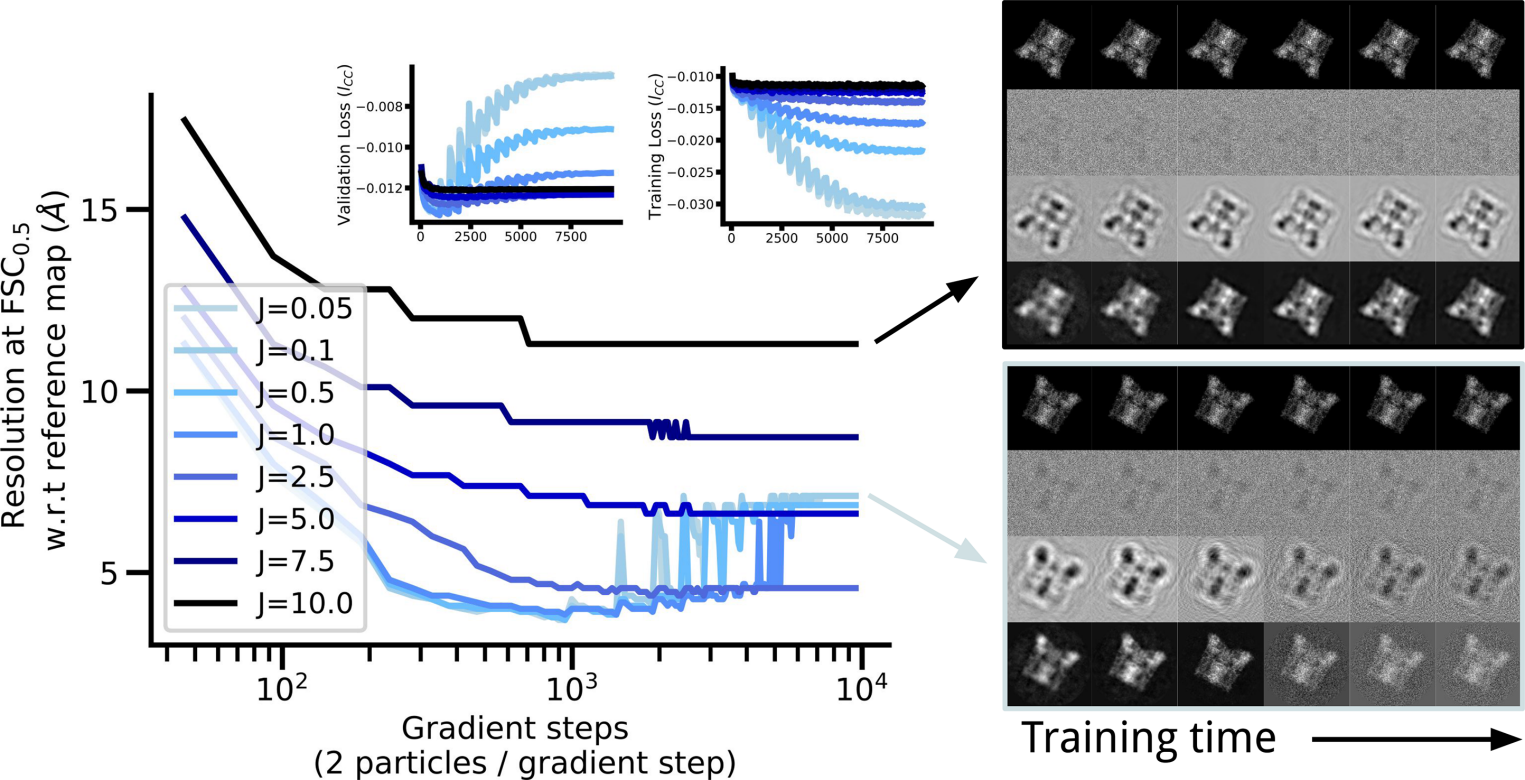
Jittering regularizes *InstaMap*. Volume resolution at FSC_0.5_ as a function of the number of particles used for training for different jittering scaling values *J*. The loss is shown as insets for the training and validation sets. With few (<1000) particles and many (>20) training epochs, *InstaMap* fits high-resolution noise. This can be ameliorated by jittering the grid and increasing its scaling *J*. This acts in a similar way as a low-pass filter, preventing both high-resolution signal and noise overfitting, as can be seen from the light-blue (*J* = 0.05) and dark-blue (*J* = 10) panels on the right for two poses. For each example pose, the four subpanels from top to bottom show the top view of the TRPV1 ion channel: projected reference volume, synthetic data point, projected *InstaMap* with CTF and projected *InstaMap*. These are shown for increasing *InstaMap* training time points at gradient steps 47, 94, 522, 4985 and 9495.

**Figure 4:**
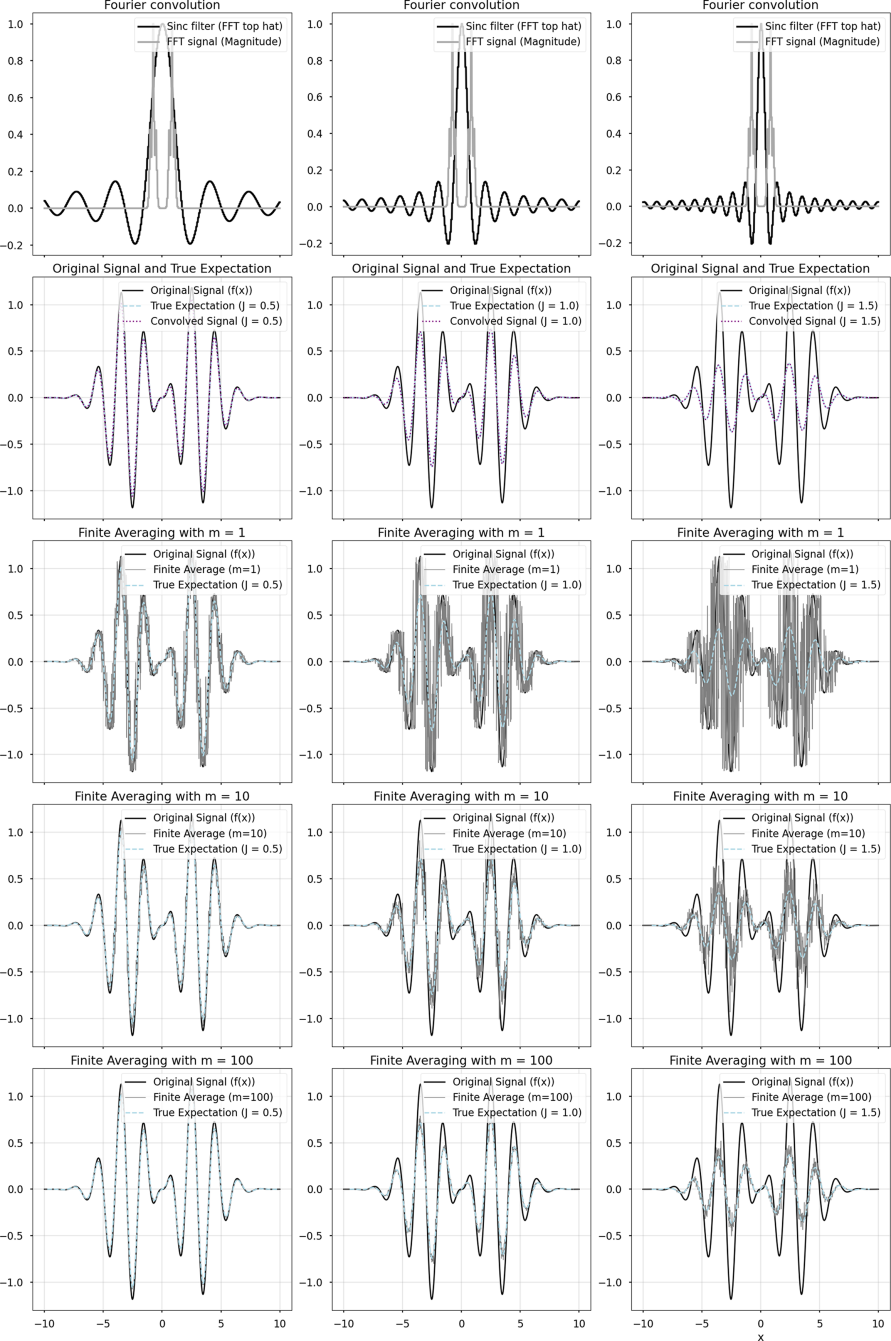
Jittering is equivalent to convolving with a sinc filter in expectation. The one-dimensional signal 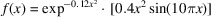 is shown for different levels of jitter *J* ∈ {0.5, 1, 1.5} and different levels of averaging {1, 5, 50, 500, 5000}. In expectation, jittering corresponds to a real-space convolution with a top-hat filter. This agrees with the equivalent operation in Fourier space via the convolution theorem, where it corresponds to multiplication by a sinc filter.

We observe that excessive epochs with small numbers of particles yields an InstaMap whose 3D renderings contain noisy artifacts that appear as ‘small dusty blobs’. We show this in Fig. 3 ▸, where with increased training time, rendered projections contain similar salt-and-pepper noise features as observable in the synthetic data, indicating overfitting to noise. We hypothesized that jittering the grid points would ameliorate this effect, at the expense of introducing disagreement in the signal, which is recovered in expectation. In Fig. 3 ▸, we quantify the trend empirically by overtraining a batch of 1000 particles at various jitter levels, *J* ∈ {0.05, 0.1, 0.5, 1.0, 2.5, 5.0, 7.5, 10.0} Å, highlighting the tradeoff between a limited resolution and the occurrence of noise artifacts. The FSC-estimated resolution increases, and this increase indicates a problematic overfitting after ∼3000 gradient steps, *i.e.* epoch 6. The rendering panel late in training for *J* = 0.05 shows noise artifacts appearing. With *J* = 10 these artifacts are absent in the projected images, but the resolution plateaus around 12 Å. For *J* ≤ 1.0 the resolution reaches 4 Å, but then increases to 7 Å. Interestingly, there seems to be an optimal level of jittering at *J* = 2.5 where the resolution remains at ∼5 Å and does not worsen. Jittering is a real-space operator analogous to the low-pass Fourier filters commonly used in cryo-EM reconstruction software: uniformly jittering coordinates in a box becomes a convolution with a box (top-hat) filter in expectation, and thus a sinc filter in Fourier space, as illustrated in Fig. 4 ▸. Although Fourier filtering could also be introduced in the input data set, or on the fly in the loss function, there is a major difference in applying jittering: it smooths out the scalar function implicit in the neural network without the need for filtering, which can be useful for super-resolution rendering or computation of numerical derivatives that do not required advanced noise stabilization (van Breugel *et al.*, 2020 ▸). Furthermore, it can be applied locally at different levels for different spatial locations. This is an example of how our real-space-based representation has advantages over the Fourier-space alternatives popularly employed.

### 3.3. A large instant-NGP hash size achieves high resolution faster

**Figure 5:**
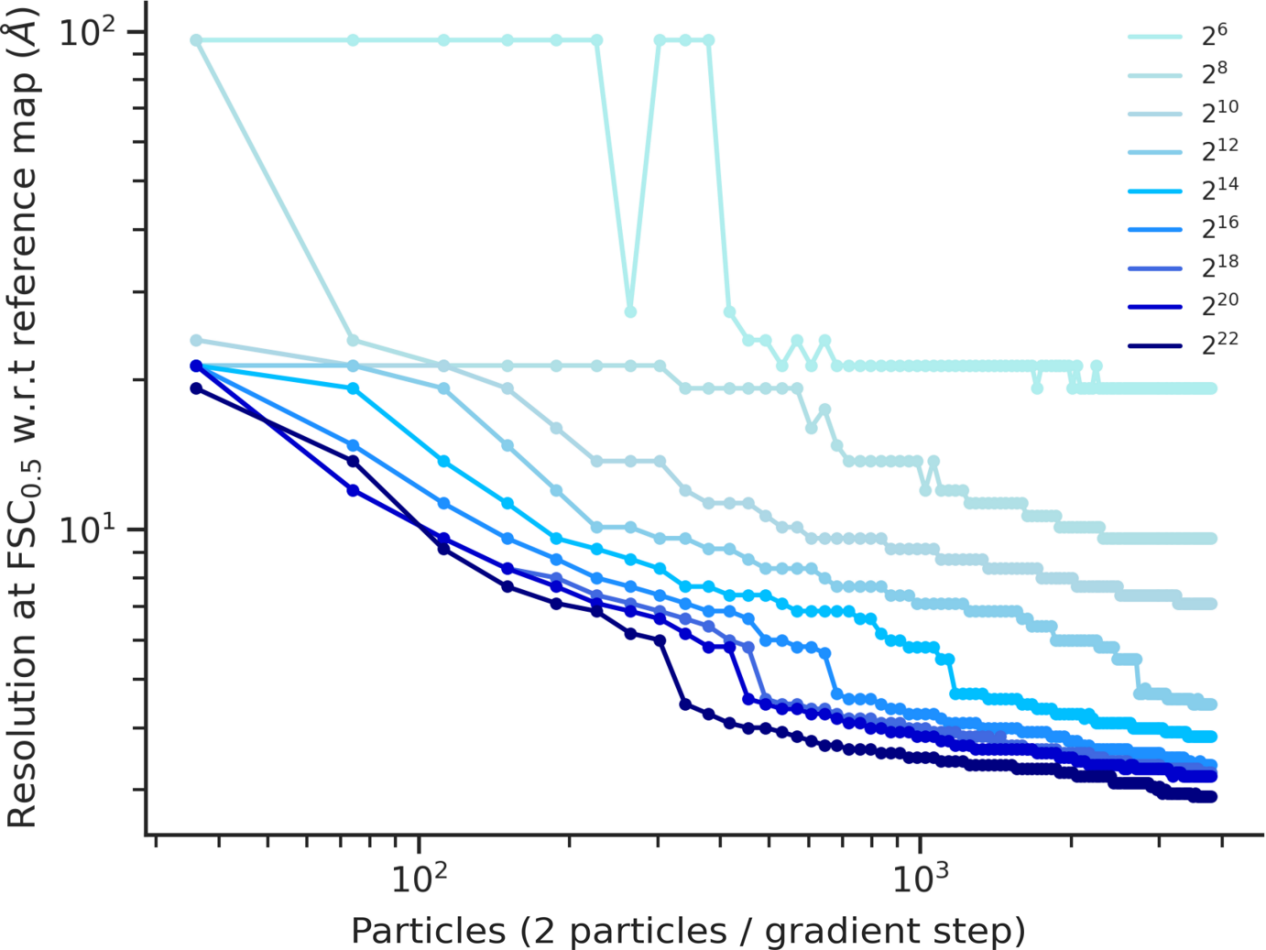
A large instant-NGP hash size achieves high resolution faster. The hash size (*F*) of instant-NGP is controlled by the log2_hashmap_size key passed in the encoder configuration. This has a large effect on the total number of trainable parameters, which is shown in Table 1 ▸.

The number of trainable parameters in *InstaMap* is controlled through a ‘Grid’ encoder and MLP decoder configuration in tinycudann, with various hash settings outlined in Section 2.3. The original authors of instant-NGP emphasized that only the hash size and finest resolution need to be tuned to the task (Müller *et al.*, 2022 ▸; Table 1 ▸). We adapted the hyperparameters of instant-NGP to our cryo-EM problem by matching the finest resolution based on the Nyquist frequency of the observations, and performed experiments on the hash size with a lightweight two-layer MLP with 64 or 128 neurons each. Fig. 5 ▸ shows that *InstaMap* with a hash-map size of *F* = 2^12^–2^22^ at *L* = 8 levels for a total number of trainable parameters of 75 000–11 400 000 (Table 1 ▸) suffices to reach FSC_0.5_ resolutions of 7–10 Å after training on a few hundred particles and up to 3–4 Å after a few thousand. However, smaller hash sizes took longer to reach higher quality resolutions and some did not reach resolutions better than 10 Å. This is not surprising since these small instant-NGP architectures contain few trainable parameters (≤35 000). Comparing the performance of a mid-sized hash map with the frequency neural scalar field and voxel representations, 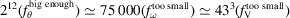, illustrates that *InstaMap* can quickly reach high resolutions with a number of trainable parameters that corresponds to a lightweight frequency neural scalar field or a real-spaced volume downsampled to a 43-pixel box size [(2^12^ = 4096) ≪ (75 000 ≃ 43^3^ = 79 507)].

**Figure 6:**
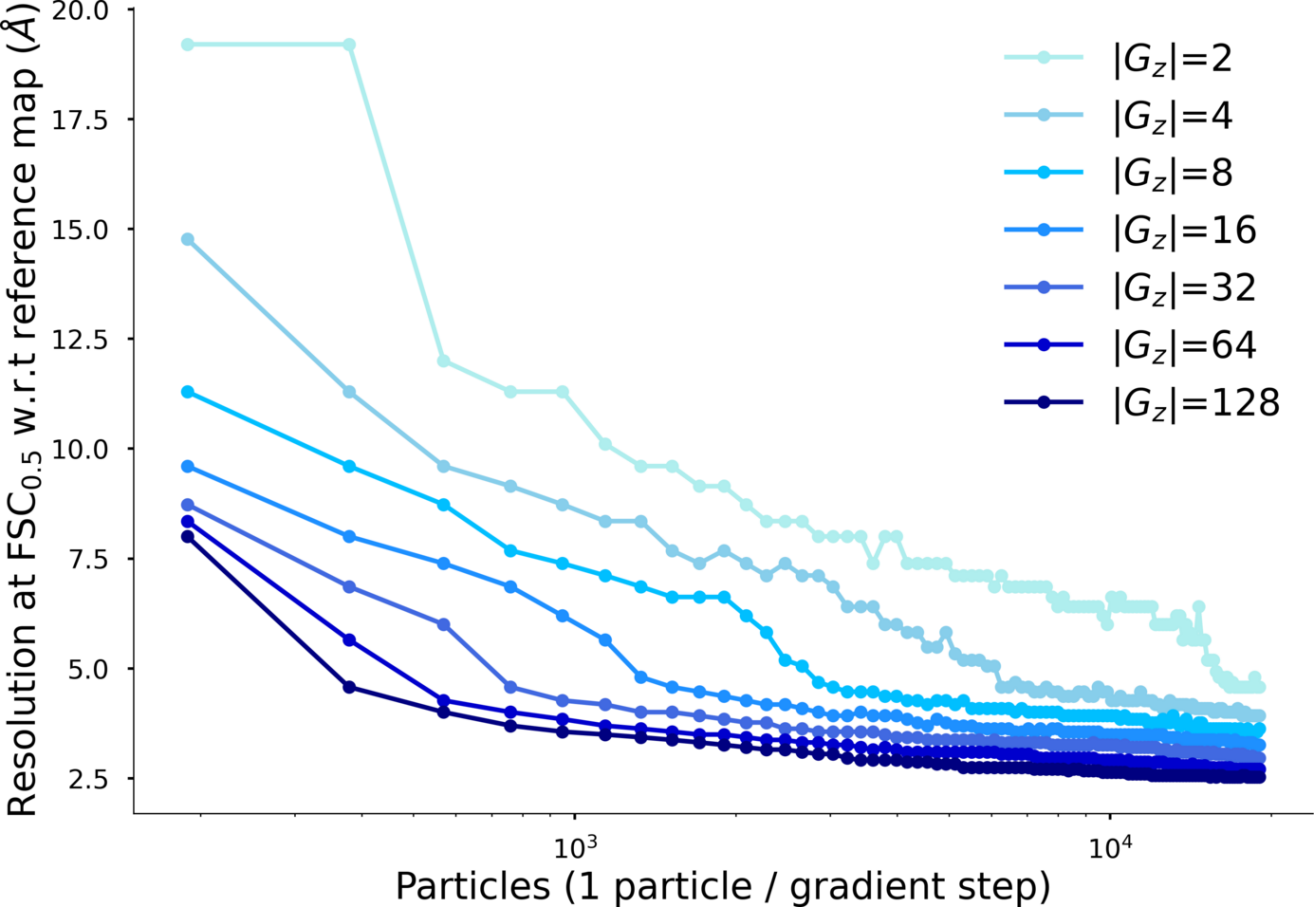
A large number of query points |*G*| reaches high resolution faster. The number of grid points in the imaging direction used to query instant-NGP is controlled by the depth_samples=|*G*_*z*_| hyper-parameter of *InstaMap*. This has no effect on the total number of trainable parameters, but rather the memory and run time.

As mentioned in Section 2.2, the number of total grid points in the viewing direction, *G*_*z*_, is a hyperparameter. It is uncoupled from the pixel size of 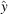. In Fig. 6 ▸ we show that *InstaMap* trains faster with more points, and we settled on using 64 or 128. Interestingly, with |*G*_*z*_| as low as 2, 4 or 8, *InstaMap* still reaches a good FSC, although there are ‘dotting’ artifacts in the rendered maps. At |*G*_*z*_| = 16, 32, 64, 128 there are no such artifacts.

### 3.4. Instant-NGP reconstructions on empirical data can achieve high resolution

Having validated our pipeline on synthetic data, we assessed the ability of *InstaMap* to analyze real experimental cryo-EM data sets: TRPV1 and apoferritin (see Section 2.6). Firstly, we compared the performance of *InstaMa*p in early training with direct Fourier inversion (DFI), a classical solution to tomographic projection implemented in many iterative refinement methods (Penczek, 2010 ▸; Glaeser *et al.*, 2021 ▸). We use DFI here following the terminology from Scheres (2012*a* ▸) to refer to algorithms that perform the merging of the 2D information from images into a 3D reconstruction in Fourier space. DFI is related to filtered back-projection and Fourier gridding, and their distinction relates to how interpolation errors are corrected for (real space or Fourier space); see the sections *Theory: Conventional Methods* and *Experimental Procedures: Implementation* in Scheres (2012*a* ▸) for more detail. We compared volumes reconstructed from two disjoint subsets of various sizes, and used the FSC tool in *RELION* (Kimanius, Dong *et al.*, 2021 ▸), reporting the frequency at which the FSC was below 0.143, as commonly used in the community (Kleywegt *et al.*, 2024 ▸). Concretely, we used relion_reconstruct to perform DFI, which resulted in maps that were sometimes visually noisy, likely due to the lack of spectral weighting (map sharpening). We used relion_postprocess to compute the FSC, which sharpens maps with a filter derived from the FSC. This experiment was performed with the TRPV1 empirical data. As expected the resolution increased with more particles (Fig. 11). To compare DFI with *InstaMap*, we independently trained two InstaMaps using the same disjoint particle half-sets and FSC_0.143_ criteria as in the DFI case. In the regime of dozens to a few hundred particles *InstaMap* reaches ∼10 Å resolution, with DFI at a similar value of ∼13 Å. One limitation of the FSC with noisy half-maps is that they may correlate with each other, but not with an accurate high-quality map. This happens when two maps experience the same bias during early stages of reconstruction, despite not being an accurate estimation of even their own final reconstruction. This is a limitation of the FSC as a validation metric. Therefore, we also compared with DFI by using the FSC with a reference map. To avoid bias, we used a reference map from DFI for the DFI FSC and a reference map from *InstaMap* for the *InstaMap* FSC. These reference maps were from an independent half-set of 47 921 particles, *i.e.* a half-map (Fig. 11). Fig. 11 shows that for small batches of particles, *InstaMap* renders maps with a similar resolution as DFI. We also note that for these small sets of particles the *InstaMap* training time (minutes to tens of minutes) is roughly of the order of that for DFI (tens of seconds to minutes), because *InstaMap* has completed only one epoch of training. Concretely, on an NVIDIA A100-SXM4-40GB GPU, *InstaMap* is able to update at the rate of 34 ± 1 particles per second for hash sizes of 2^12^–2^22^ for TRPV1 synthetic data. For a run time of 10 min, this corresponds to analyzing 20 400 ± 600 particles. Most experiments were performed in one epoch. For 100 000 particles this run time corresponds to 49 min. These run-time estimates neglect any computing validation metrics.

The artificially high-resolution FSC between instant-NGP half-maps is due to them training in a similar way, indicating a similar inductive bias that carries correlating high-resolution detail. When we compare the FSC with the reference, it gradually improves and does not have any early training artifacts. This result cautions against naïvely relying on the FSC between half-maps as a validation metric when there is relatively strong inductive bias in the volume representation.

**Figure 7:**
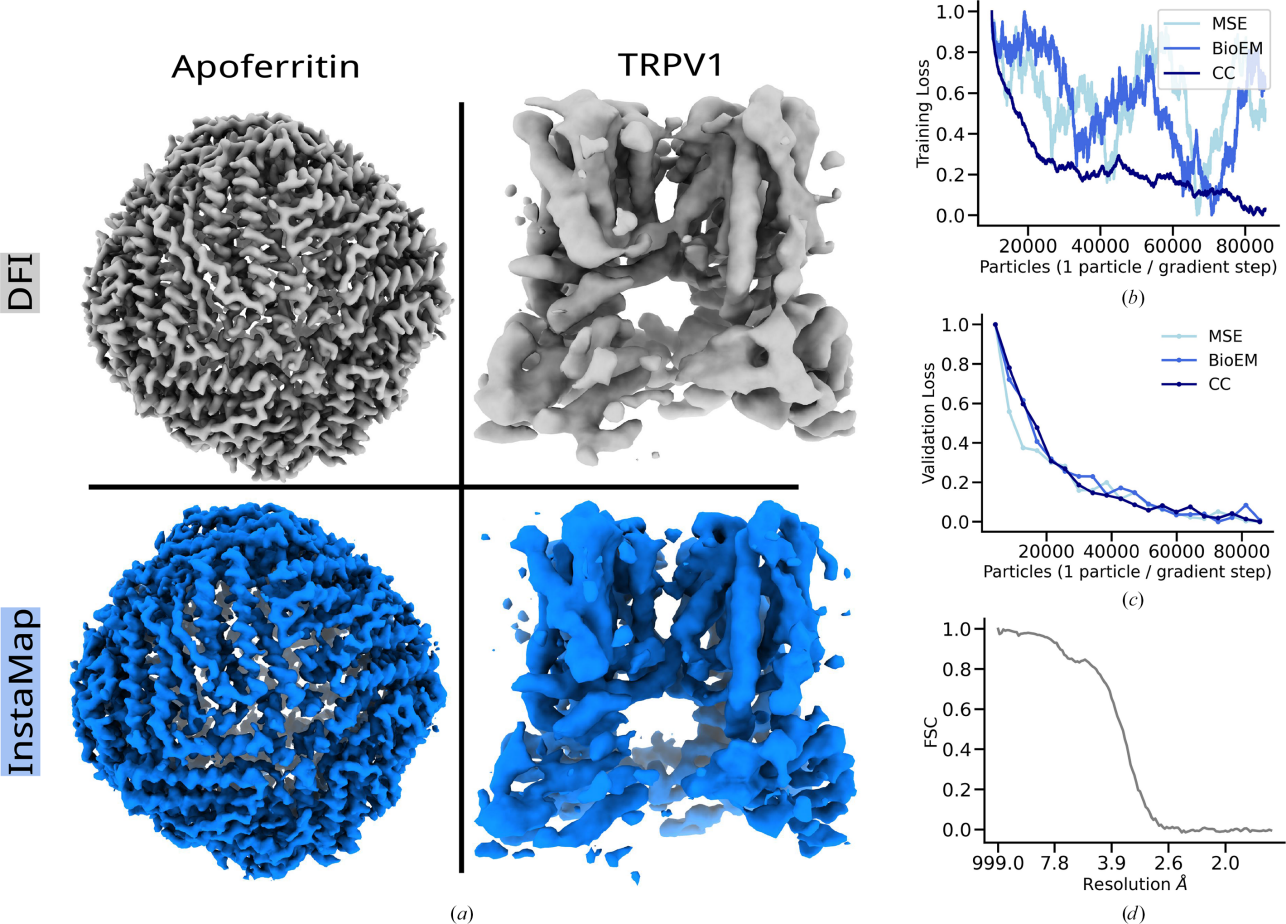
Instant-NGP reconstructions of empirical apoferritin and TRPV1 cryo-EM images can achieve high-resolution details. (*a*) Direct Fourier inversion (DFI) and *InstaMap* reconstructions (with *l*_CC_) are shown side by side for visual inspection. Both methods used a similar number of particles (DFI, 80 000 × 2 for two half-maps; *InstaMap*, 80 000). (*b*, *c*) Reconstructions with each loss function (*l*_CC_, *l*_MSE_, *l*_BioEM_) were performed and are shown as a function of training time for apoferritin. The training loss was smoothed with a running average of size 10 000. The noise level σ = 3 was assumed for *l*_CC_ and *l*_MSE_, but note that it is merely a multiplicative scaling. (*d*) The FSC of apoferritin is computed between the DFI and *InstaMap* maps that are shown.

We then performed reconstruction experiments on both TRPV1 and apoferritin empirical data sets and reached a similar resolution compared with DFI. Fig. 7 ▸(*a*) shows both specimens aligned side by side for visual comparison of fine details and artifacts. The three graphical panels in Figs.7 ▸(*b*)–7 ▸(*d*) show the loss over the course of training for training (Fig. 7 ▸*a*) and test (Fig. 7 ▸*b*) sets, as well as an FSC between the DFI and *InstaMap* volumes for apoferritin (Fig. 7 ▸*c*). The three loss curves (scaled between 0 and 1) for *l*_CC_, *l*_MSE_ and *l*_BioEM_ show a similar trend for decreasing validation loss. However, *l*_CC_ shows much less variance during training, which is likely due to the invariance to multiplicative and additive global scaling from *w*. This is often a desirable property, and thus further investigation is merited, especially with empirical data, which can contain large outliers due to latent variable inaccuracies (pose, CTF *etc.*) or ‘junk particles’. We thus used *l*_CC_ for all other homogeneous reconstruction experiments.^2^ We note that the losses decreased more slowly for empirical data compared with synthetic data, which is not surprising given that empirical data have different noise statistics compared with the Gaussian white-noise model that we used to generate synthetic data. Notably, *InstaMap* trained on 80 000 particles reached a similar level of visual detail as in DFI (after filtering in *RELION* post-processing tools via two half-maps of 80 000 particles), suggesting that the resolution is limited by the empirical data (pose-estimate accuracy, heterogeneity and noise).

Interestingly, even though the volume from *InstaMap* has not been spectrally filtered, it is very similar in appearance with minimal high-frequency noise, which is likely to depend on the power spectra of the input particles, which decayed at high frequency in these EMPIAR particles stacks, although this is not always the case in EMPIAR.

### 3.5. Masking via subsetting coordinates

**Figure 8:**
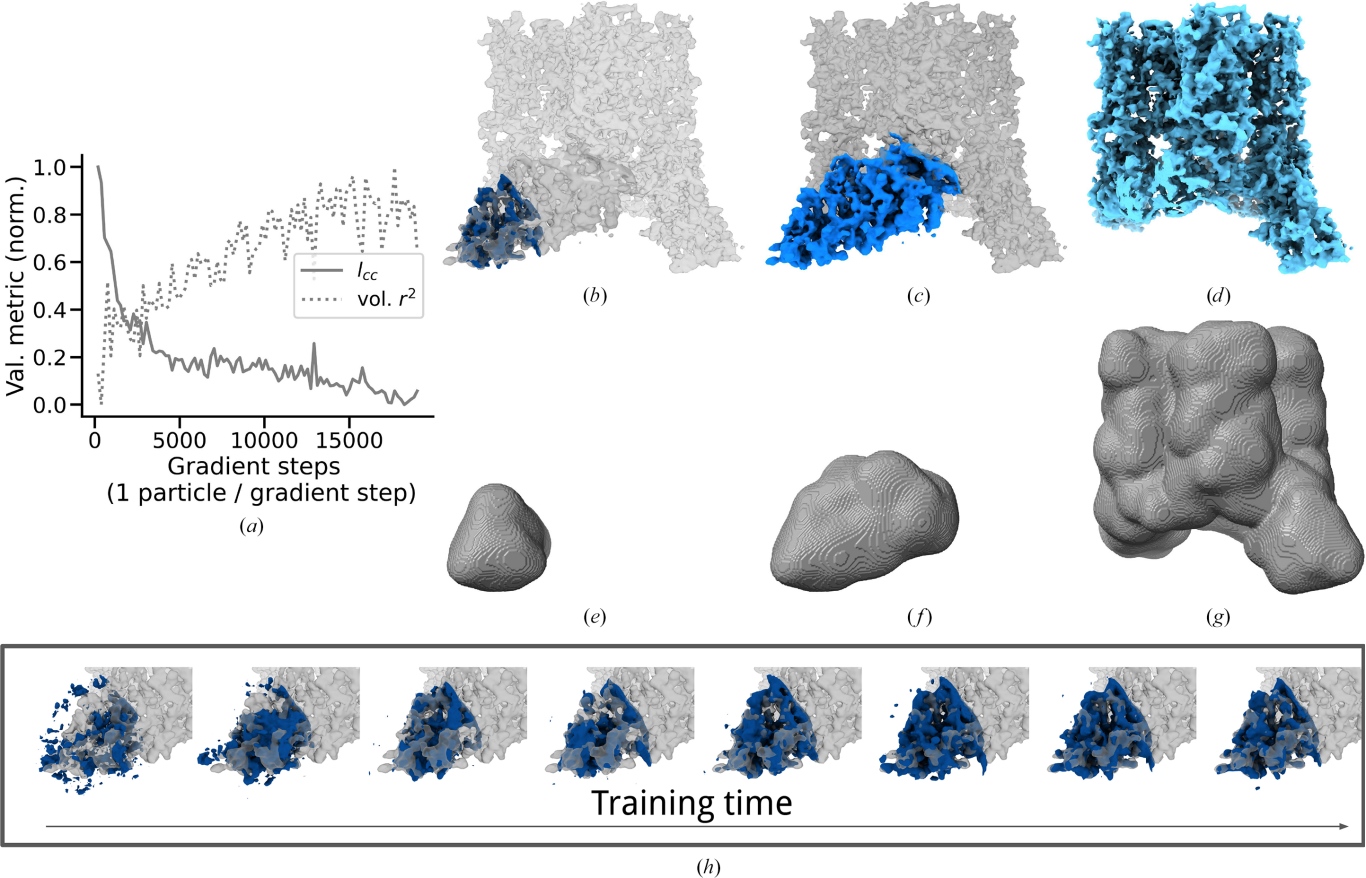
Masking preserves most detail. Loose masks were constructed with our own implementation of cosine filtering after segmentation in *Chimera* (Meng *et al.*, 2023 ▸) with *Segger* (Pintilie, 2010 ▸). Small (*e*), medium (*f*) and large (*g*) masks were created, with density reconstructed in dark blue (*b*), aqua (*c*) and light blue (*d*), respectively. (*b*) Reconstructed density inside the small mask is colored dark blue, with density from the other regions shown in gray (darker gray for medium, lighter gray for large). (*c*) Reconstructed density inside the medium mask is colored blue, with density from the large region in gray. (*d*) Reconstructed density inside the large mask is colored light blue. For the smallest mask, the validation loss in image space (*l*_CC_) and volume (real-)space (Pearson correlation, volume *r*^2^) improve over training (*a*), as shown visually (*h*) for gradient steps 10, 30, 100, 300, 1000, 3000, 10 000 and 180 000.

We masked by restricting the pose-specific query points *G*_*i*_ to fall inside a mask. An arbitrarily shaped mask is provided as an input file (float datatype) and linearly interpolated at the pose-specific query points. The grid points above some cutoff (in our experiments we used zero) are cast to a Boolean datatype, *i.e.* binary mask. The pose-specific projection matrix (*M*) is formed for the subset of grid points that fall within the mask. Fig. 8 ▸ shows a slight loss of detail as the mask is made smaller and also shows how detail emerges during the optimization (training). In particular, note how the smallest reconstructed subvolume (Fig. 8 ▸*b*) fills out the medium reconstructed region;also note the similar quality of features in the medium (Fig. 8 ▸*c*) and large (Fig. 8 ▸*d*) regions. One of the challenges of validating small masked regions with the FSC is that regions at the boundary of the mask have sharp transitions that carry high-frequency content; thus, when small regions share this boundary they can correlate at high frequency despite dis­agreeing in the interior. To avoid this confounding phenomenon we looked at non-FSC validation metrics. Fig. 8 ▸(*a*) shows an increase in real-space 3D volume correlation, decreasing validation loss (*l*_CC_), and better visual detail (Fig. 8 ▸*h*) over training time for a small region. Note that soft masks can be used in our approach, which avoid a sharp boundary.

### 3.6. Inferring heterogeneity via bending space

In contrast to cryo-EM heterogeneity methods that displace mass (Zhong, Lerer *et al.*, 2021*b* ▸; Rosenbaum *et al.*, 2021 ▸; Chen & Ludtke, 2021 ▸; Chen *et al.*, 2023 ▸; Schwab *et al.*, 2024 ▸; Vuillemot *et al.*, 2023 ▸), here we bend space. This distinction is made in literature on point-based rendering/splatting (Kopanas *et al.*, 2021 ▸; Kerbl *et al.*, 2023 ▸). The fluid dynamics community has developed related terminology in the form of two modelling traditions for flow fields: (i) the Lagrangian, which tracks pieces of mass through trajectories in a velocity field and is often simulated mesh-free (displaces mass), and (ii) the Eulerian, which focuses on fixed points in space through which material flows and often uses a fixed mesh (bends space; https://en.wikipedia.org/wiki/Lagrangian_and_Eulerian_specification_of_the_flow_field). *InstaMap* models heterogeneity through deforming the spatial queries into the globally learned reference field (line 12 in Algorithm 1). Our approach is similar to how space is bent in another cryo-EM heterogeneity inference method, *Zernike*3*D*: see equations 4, 5 and 9 in Herreros *et al.* (2023 ▸). Bending space, as we employ this term, is ‘image warping’ from digital image processing, where there is a spatial transformation that warps coordinates to produce mappings between input and output images. Standard treatments distinguish forward and reverse mapping (see Sections 3.1.1 *Forward Mapping* and 3.1.2 *Reverse Mapping* in Wolberg, 1994 ▸). In forward mapping, the warping function sends input coordinates (and the respective intensity at this coordinate) to output coordinates, and requires accumulators or interpolation to resolve issues with clashes and holes. Reverse mapping applies a function to output coordinates, such that the inverse coordinate (and its associated scalar) are queried. Whereas both Herreros *et al.* (2023 ▸) and Punjani & Fleet (2023 ▸)^3^ apply forward mapping, here we apply reverse mapping.

**Figure 9:**
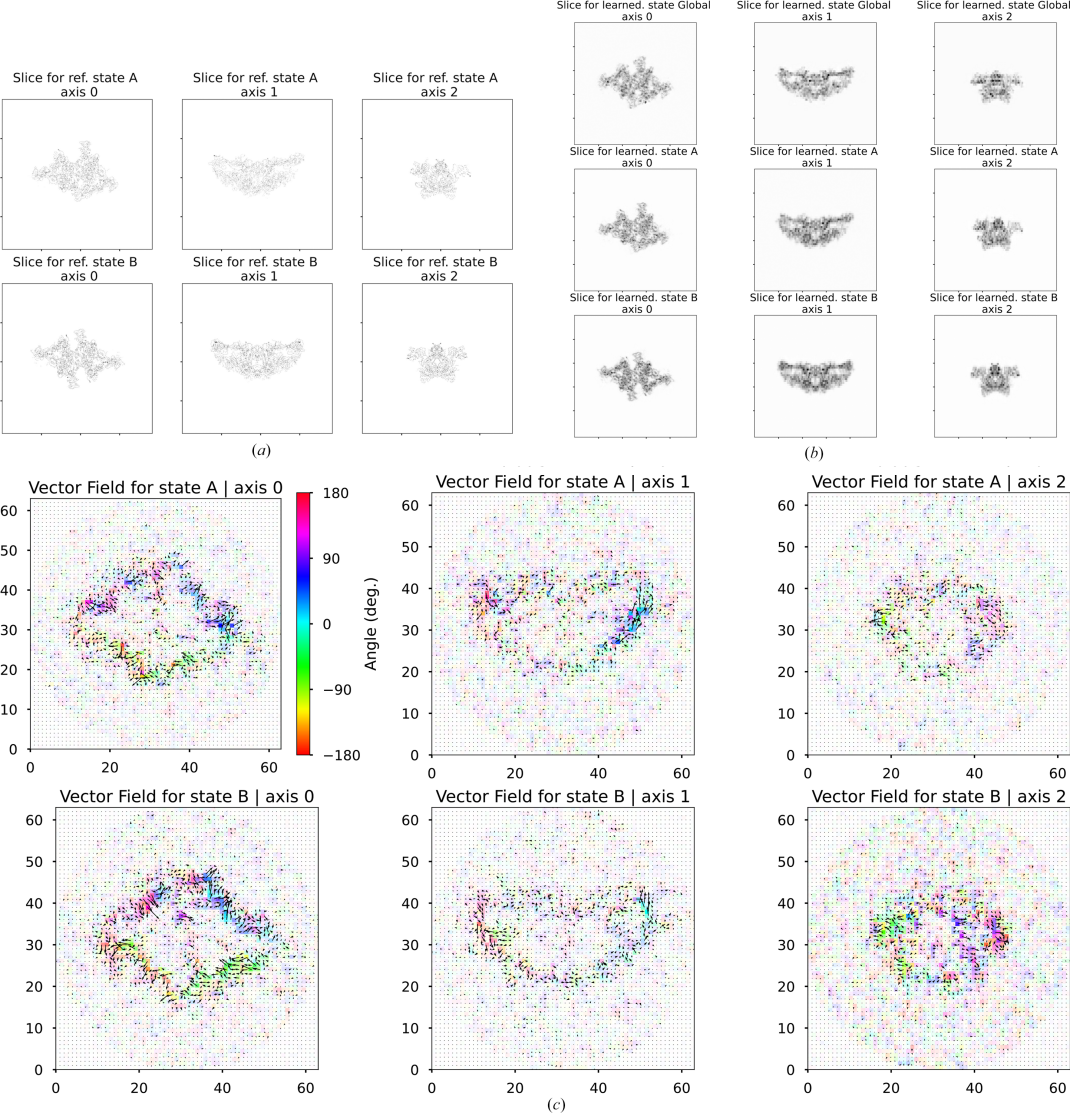
Heterogeneity via bending space. Two reference volumes of thyroglobulin (*a*) are similar to their inferred heterogeneous volumes (*b*). (*c*) Heterogeneity is visualized from 1000 averaged vector fields conditioned on their predicted class (as determined by clustering the vector-field cosine similarity matrix; see Section A7) and shown as a 2D slice averaged from the middle eight voxels. The magnitude is visualized as opacity and the angle as color; they are overlaid with a quiver showing the direction.

**Figure 10:**
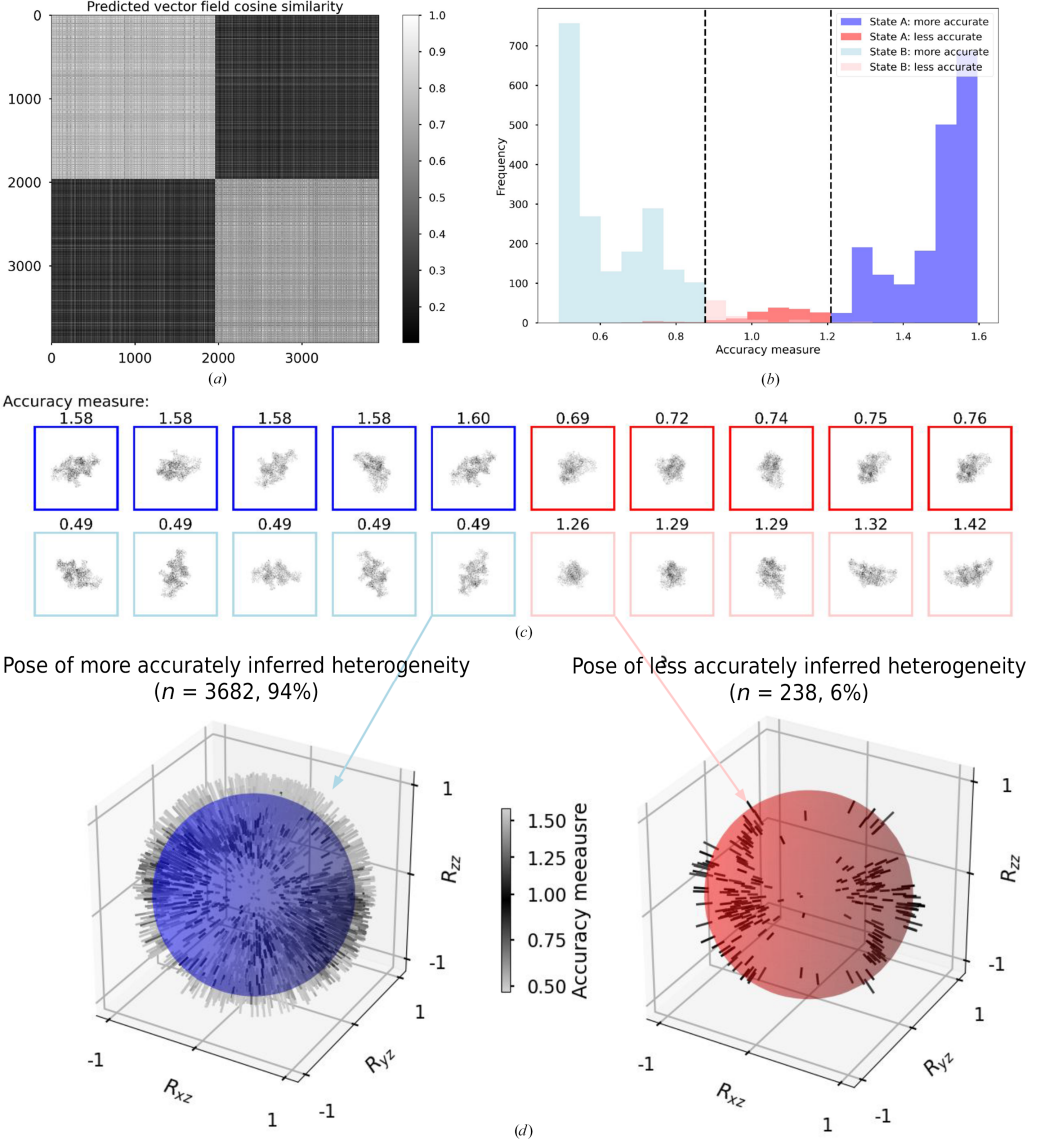
Entanglement of heterogeneity vector fields with pose. Inaccuracies are entangled with pose due to information loss of the projection of density from 3D → 2D. After training, vector fields for (test-set) images from two thyroglobulin states were inferred from their respective images (1960 in state A; 1960 in state B). (*a*) Cosine similarity between predicted latent variables (predicted per-image vector field and predicted per-image density) is significantly higher within states (0.75 ± 0.20, 0.66 ± 0.23) than between states (0.30 ± 0.12), as indicated by the block structure. (*b*) The distributions of the accuracy measure, conditioned on ground-truth heterogeneity, are not completely separated, and we show the 6% overlapping region in red (dark red for state A, light red for state B). (*c*) Images with more accurately inferred heterogeneity (blue) have a pose that reveals more about the conformational heterogeneity (see Fig. 9 ▸*a*; axis 0), while images in the less accurate region (red) have a view that obscures the conformation (see Fig. 9 ▸*a*; axes 1 and 2). (*d*) The pose conditioned on inferred heterogeneity shows the entanglement: the pose distribution of images with more accurately inferred heterogeneity is different from the pose distribution of less accurately inferred heterogeneity. Thus, while the ground-truth distribution of pose and heterogeneity are independent, the inferred heterogeneity is entangled with pose.

Heterogeneity via bending space was implemented as a vector field. We ensured SE(3) equivariance through outputting the vector field onto a regular grid in a canonical frame. We then query the canonical vector field at the same rotated and translated set of points that it linearly preturbs. The perturbed points query the global scalar density in a reverse mapping manner; see equations (7) and (8). This was inferred per image via amortized inference, where global parameters φ in *F*_φ_ are shared between all images, and the output is estimated for each image. Fig. 9 ▸ shows the heterogeneity of thyroglobulin simulated data, taken from the course-grained representation of the motion underlying the Inaugural Flatiron Heterogeneity Challenge (Astore *et al.*, 2023 ▸). Fig. 10 ▸(*a*) shows that the inferred vector fields are similar between images from the same heterogeneity state. Inaccurate inference of heterogeneity also arises when the shape difference is obscured by the pose (Fig. 10 ▸*b*), as expected due to the loss of information by projection. Solutions to the entanglement of pose and shape have been explained and studied (Klindt *et al.*, 2024 ▸), and a disentanglement algorithm has recently been implemented in 3D reconstruction (Herreros *et al.*, 2024 ▸) for pose and CTF. Applying this type of disentanglement regualrizer in *InstaMap* would involve repeated queries of 3D coordinates into instant-NGP, which can be performed in batch with sufficient memory resources.

## 4. Discussion and conclusions

### 4.1. Overview of our contribution

*InstaMap* adapts the multi-resolution hash instant-NGP to the cryo-EM inverse problem. A main advantage of *InstaMap*’s multi-resolution hash are fast training and the absence of confounding artifacts. Our comparisons with other scalar density parametrizations (*f*_TW_, *f*_SH_, *f*_OB_ and *f*_ω_; with approximately the same number of trainable parameters, 200 000) and voxel intensity (∼20× more trainable parameters, 4 million) show that a medium-resolution rendering can be produced after learning from 50–100 particles. While other methods can have artifacts (for example dusty noise, a meshy grid or ‘hedgehog spikes’), the renderings from *InstaMap* appear gently blurred out, which is expected from the multi-resolution hash. Structural biologists frequently have to interpret the biochemical meaning of densities at the limit of reliability, often working in regimes where these artifacts are present (Lawson *et al.*, 2021 ▸, 2024 ▸; Lander, 2024 ▸). While the voxel intensity representation has more parameters, it is challenging to reach high resolution in cryo-EM with first-order gradient-based methods. In this context, recent work shows that an efficient preconditioner can accelerate convergence to high resolution via stochastic gradient descent (Toader, Brubaker *et al.*, 2023 ▸), showing benefits to representing the object in Fourier space and benefits over AdamW, the first-order optimizer we used. In our comparisons, we took care to employ a frequency neural scalar field of comparable size to *InstaMap*, but direct comparisons with previously published work (Donnat *et al.*, 2022 ▸; Toader, Sigworth *et al.*, 2023 ▸) should take into account their larger architecture (and therefore better expected performance), or even be based on memory or run-time bottlenecks rather than number of trainable parameters. For instance, the recently published *cryoSTAR* used a volume render comprised of a five-layer MLP with 32, 64, 128, 256 or 512 ReLUs (Li *et al.*, 2024 ▸). This was in continuity with methods such as *cryoDRGN* and related methods (Zhong, Bepler *et al.*, 2021 ▸; Levy, Raghu *et al.*, 2022 ▸; Levy, Grzadkowski *et al.*, 2024 ▸). However, with the recent availability of common data sets and validation metrics for synthetic ground truth (Jeon *et al.*, 2024 ▸) and blinded community challenges (Astore *et al.*, 2023 ▸), it seems a suitable time to compare neural network architectures by diverse measures of performance.

### 4.2. Comparison with the current state of the art

When veteran experts in the field gave us feedback on *InstaMap*, they raised the legitimate question of its similarities, differences and benefits over the current state of the art. In this section, we provide a reflection based on the dictum *respice*, *adspice*, *prospice*: look to the past, look to the present, look to the future. Our overall goal in this project was to adapt a neural implicit representation of the volume to cryo-EM reconstruction via end-to-end gradient-based learning, and this involves several conceptual differences from current state-of-the-art cryo-EM reconstruction pipelines, which we now attempt to distinguish.

In order to meaningfully engage in this question with clarity and precision, some distinctions are in order. First off, what is meant by current state-of-the-art approaches? While expectation-maximization solutions to maximum-likelihood or maximum-*a posteriori* objectives remain an integral part of state-of-the-art pipelines (Tang *et al.*, 2007 ▸; Punjani *et al.*, 2017 ▸; Grant *et al.*, 2018 ▸; Kimanius, Dong *et al.*, 2021 ▸), gradient-based methods that are often parametrized by differential programming approaches (*i.e.* deep learning and neural networks) are also quite popular, in particular gradient-descent methods in *ab initio* reconstruction.

Another distinction is numerically employing the Fourier slice-projection theorem to invert images into a 3D volume via filtered back-projection (FBP) or DFI of CTF-corrected images. Indeed, it is one thing to leverage the analytical inversion of the forward model via slice insertion, and another to optimize global variables that implicitly define the volume [for example with gradient-based methods as in Nashed *et al.* (2021 ▸), Kimanius *et al.* (2022 ▸) and Shekarforoush *et al.* (2024 ▸)]. The nuance with this distinction is that these gradient-based methods are using the Fourier slice-projection theorem in the differentiable forward model, and updating the gradient based on matching the ‘clean signal’ of the per-measurement (pose, heterogeneity) volume representation with the observed image. We note that inference via gradient-based optimization could still be performed with more complicated (but still differentiable) forward models of scattering (for example multislice; Kirkland, 2020 ▸; Himes & Grigorieff, 2021 ▸; Parkhurst *et al.*, 2021 ▸; Nguyen *et al.*, 2024 ▸) where inverting 3D → 2D scattering via 2D → 3D slice insertion does not hold.

The term ‘end-to-end’ typically emphasizes the lack of feature extraction from raw input data; see the introduction and related work section of Mukherjee *et al.* (2021 ▸) for end-to-end reconstruction in inverse problems in the context of data-driven regularization. As alluded to above, a major benefit of end-to-end gradient-based approaches is that the forward model (from latents to observed variable) of image formation can be made complex yet tractable, without being concerned with explicitly inverting the model (from observed variable to latents). In this work, we did not ‘undo’ the heterogeneity in each image, and then average them together via DFI, although end-to-end methods have been combined with inversion to improve high-resolution features under continuous deformations in the promising and thoughtful work of Schwab *et al.* (2024 ▸). In our case, while the addition of the vector field to the grid coordinates is invertible (it can be subtracted; see line 8 in Algorithm 1), this can be relaxed to have a richer relationship. This raises the question of what types of forward models are desirable, and how their richness and flexibility effects their interpretability and uniqueness, and numerical trade-offs.

End-to-end approaches can be combined with symbolic/algebraic inversion/manipulation of the bespoke cryo-EM forward model, for instance in our modification of the BioEM loss function in Sections 2.5 and A4. There we marginalized out unknown parameters in the likelihood. The end-to-end optimization of the scalar density, including its overall magnitude, introduced a numerical issue that drove the magnitude to zero under this loss. We solved this by re-deriving it under a broad Gaussian centered at a numerically stable (nonzero) value.

In a comprehensive survey of methods using data-driven perspectives to solve inverse problems, Arridge *et al.* (2019 ▸) distinguish various forms of regularization: (i) approximate analytic inversion (for example filtered back-propagation for computerized tomography and cryo-EM), (ii) iterative methods with early stopping, (iii) discretization as regularization and (iv) variational methods. Here, we did not use the approximate analytic inversion of DFI after CTF correction, which corresponds to regularization form (i). We also showed that regularization by real-spaced jittering allowed us to avoid some artifacts, and not tune the number of training epochs for early stopping (ii), as shown in Fig. 3 ▸. The discretization of *InstaMap*’s heterogeneity vector field (*F*_φ_) is regularized by having nearby coordinates (in our experiments on the order of several ångströms) be close due to linear interpolation, thereby promoting smoothness (iii). Finally, explicit regularizers on the global volume or vector field are an example of (iv), although we did not employ them in this work.

### 4.3. Alternative representations of heterogeneity

In various cryo-EM methods, the representation of heterogeneity is intimately coupled to the representation of volume (Donnat *et al.*, 2022 ▸; Toader, Sigworth *et al.*, 2023 ▸), which can be voxelized or coordinate-based and employ learnable (for example neural) or fixed (for example Fourier, Zernike) bases. In our work, the volume is treated as coordinate-based with global variables shared by all of the observations, and we bend space rather than displace through modelling a vector field that we interpolate from a regular grid onto a rotated and translated set of points. Future work could include priors/regularizers on the vector field, suitable for the union of rigid-body transformations from different domains and secondary-structural elements (Koo *et al.*, 2023 ▸), and near-incompressibility (Punjani & Fleet, 2023 ▸). Here, we have used an additive transformation to perturb the coordinates, essentially bending space along straight lines, but in future work it could be bent along more structured trajectories such as curves or parametric transformations on coordinates. We also chose to estimate a vector field per image, but it could instead be a global variable, with a per-image coordinate estimated. Inference via amortization with a neural encoder versus per-image optimization of vector field are not mutually exclusive. Amortized inference could be used to initialize the optimization of a local per-image vector field latent, as has recently been performed with pose in a two-stage manner which improved accuracy and showed the accuracy limits of amortization with a neural encoder (Shekarforoush *et al.*, 2024 ▸).

Our method could be extended to compositional heterogeneity by training multiple scalar volume fields (similar to Levy, Radhu *et al.*, 2024 ▸), and perhaps by applying bespoke disjoint masks. However, to employ an implicit neural density and then attempt to model conformational heterogeneity via displacing mass rather than bending space seems awkward to implement in *InstaMap*. Concretely, how would one displace the *mass* from 3D grid points (which depends on their real-spaced coordinate query into the implicit volume), and also move their *location* to be on the set of fixed 3D → 2D projections, in a *differentiable way* that respects pose? While it may seem feasible in a dense way, we think that more thought is required on how to maintaining differentiability while employing sparse data structures to achieve small memory allocations.

Encouragingly, significant advances rendering dynamic scenes with NeRFs have been made. Techniques such as *D-NeRF* (Pumarola *et al.*, 2021 ▸) and *HyperNeRF* (Park *et al.*, 2021 ▸) approach scene dynamics by treating deformation as an auxiliary field, akin to an Eulerian representation, where changes are mapped relative to fixed spatial points. In contrast, *ModalNeRF* (Petitjean *et al.*, 2023 ▸) adopts a fundamentally different perspective by employing a Lagrangian representation, viewing motion through the lens of particle-based fields. This method notably applies modal analysis to capture the intrinsic oscillations of objects, making it uniquely suited for the complex and nonrigid nature of proteins and biomolecules in cryo-EM studies.

### 4.4. Extending to joint estimation of pose

As a proof of concept of the benefit of using instant-NGP, *InstaMap* exclusively focuses on volume inference. We assumed that the input data were annotated with accurate estimates of pose and microscope parameters. The next natural step would then be to extend the framework to pose inference.

Extending the current work to jointly infer the pose (*i.e. ab initio* reconstruction) or pose refinement could be performed by several inference methods, such as (i) amortized inference of the pose from 2D images through training a network to predict the rotational element ∈ SO(3) or full pose ∈ SE(3) as has been performed by multiple methods (Nashed *et al.*, 2021 ▸, 2022 ▸; Koo *et al.*, 2023 ▸; Levy, Poitevin *et al.*, 2022 ▸; Levy, Wetzstein *et al.*, 2022 ▸), (ii) dictionary learning where the explicit index of each observation is associated with an inferable pose (see the commentary in Edelberg & Lederman, 2023 ▸), (iii) an explicit search strategy such as branch-and-bound (Punjani *et al.*, 2017 ▸; Zhong, Lerer *et al.*, 2021 ▸) and (iv) search and Bayesian marginalization with repeated likelihood evaluations (Scheres, 2012*b* ▸; Cossio *et al.*, 2017 ▸; Tang *et al.*, 2024 ▸), and more recently performant use of search or amortized inference with a final stages of gradient descent (Levy, Grzadkowski *et al.*, 2024 ▸; Shekarforoush *et al.*, 2024 ▸). Perhaps the feasibility of the specific inference method depends on the actual specimen being estimated. In our case, given the current PyTorch implementation of Algorithm 1, multiple evaluations of *f*_θ_ at latent poses for one observation may result in a significant memory demand, although querying of *f*_θ_ can be performed sequentially in a large batch to keep memory demand low, as there are other (run-time) computational bottlenecks in Algorithm 1 that are less memory-intensive. Furthermore, in the current iteration of *InstaMap*, heterogeneity implicitly refers to one reference map (*i.e.* when *F*_φ_ = 0). Therefore, it seems reasonable that poses could first be roughly inferred via consensus reconstruction and then be iteratively refined by predicting a pose residual by various methods.

In contrast to the approaches outlined above, employing classical computer-vision methods such as *Structure from Motion* (*SfM*) and newer techniques of joint optimization of poses and neural fields could pose a challenge. For example, traditional approaches, such as *COLMAP* (Schönberger & Frahm, 2016 ▸), are designed for scenarios where images have easily detectable and overlapping features, a condition that is not met by cryo-EM images. Similarly, cutting-edge trends in combining pose estimation with neural radiance face significant hurdles. Methods such as *BARF* (Lin *et al.*, 2021 ▸), for instance, require precise starting points for effective application, a requirement that is often unfeasible in the context of cryo-EM data. Generative and adversarial-based techniques, such as *GNeRF* (Meng *et al.*, 2021 ▸) and *VMRF* (Zhang *et al.*, 2022 ▸), typically require large data sets and complex training processes. These methods hinge on the assumption that the distribution of poses follows certain predictable patterns, an assumption that may not hold true in cryo-EM. Two methods that are particularly applicable to cryo-EM data are worth noting: *MELON* (Levy *et al.*, 2023 ▸) and *LU-NeRF* (Cheng *et al.*, 2023 ▸). Both methods group images into subgroups in an initial phase. *MELON* divides the latent space into subsets of equivalence classes, each supervised by ground-truth data, while *LU-NeRF* groups images into subgroups, employing self-supervised deep features for this initial categorization. Further empirical demonstrations are required to determine whether any of these approaches sufficiently align with the data characteristics and forward model structure in cryo-EM.

### 4.5. Future outlook

The promise of using existing solutions of the neural implicit ecosystem to incorporate pose estimation and dynamics into the *InstaMap* framework established here speaks to its potential as a viable solution as an end-to-end framework for problems in cryo-EM.

Can we directly obtain atomic models from 2D images? On the forward model/representation side, the recent advances of Gaussian splatting for scene rendering (Westover, 1991 ▸; Kerbl *et al.*, 2023 ▸) are a clear future direction of this framework as this should facilitate the direct fitting of atomic coordinates rather than volume densities. Atomic models as the underlying latent are a strong inductive bias: domain knowledge about the underlying specimen and its dynamics from biomolecular simulation and quantum chemistry sets cryo-EM (and all of structural biology) apart as a privileged inverse problem. New methods employing coordinate representations in some manner have already revealed insights on empirical data sets that infer directly on 2D image data, synthetic (Nashed *et al.*, 2022 ▸; Koo *et al.*, 2023 ▸) or empirical (Chen & Ludtke, 2021 ▸; Chen *et al.*, 2023 ▸; Li *et al.*, 2024 ▸; Schwab *et al.*, 2024 ▸; Dingeldein, Silva-Sánchez *et al.*, 2024 ▸). These studies testify to the interest and promise in incorporating atomic coordinate information, and are thanks to pioneering work (Kimanius, Zickert *et al.*, 2021 ▸; Zhong, Lerer *et al.*, 2021*b* ▸; Rosenbaum *et al.*, 2021 ▸) that was candid on the challenges experienced.

**Figure 11:**
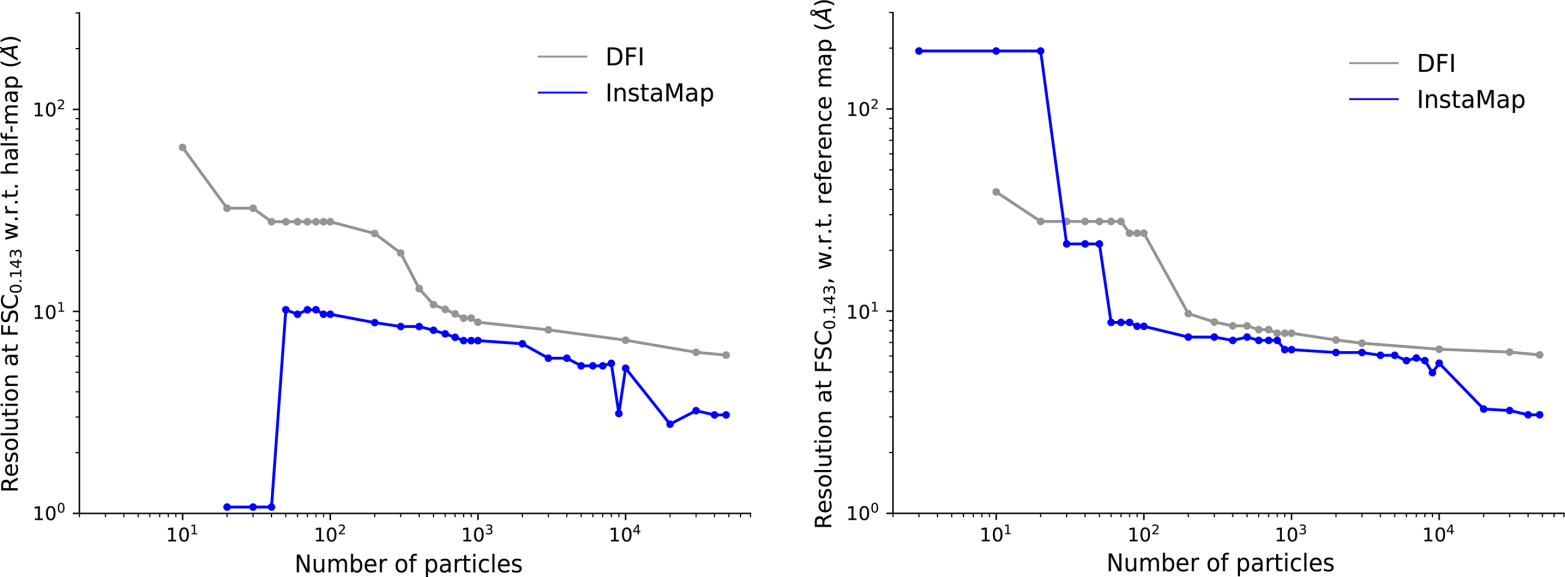
*InstaMap* achieves better resolution during early training than back-projection for empirical data. During early stages of training, *i.e.* for few images, *InstaMap* reconstructions have a better FSC than DFI. We reconstructed half-maps from two disjoint sets of particles of TRPV1 (EMPIAR 10005) with *InstaMap* (two independent training runs on disjoint half-sets of particles; blue) and DFI via relion_reconstruct (gray) and estimated their FSC via relion_postprocess. The reference maps were from the independent half-map from DFI or *InstaMap*, respectively.

**Figure 13:**
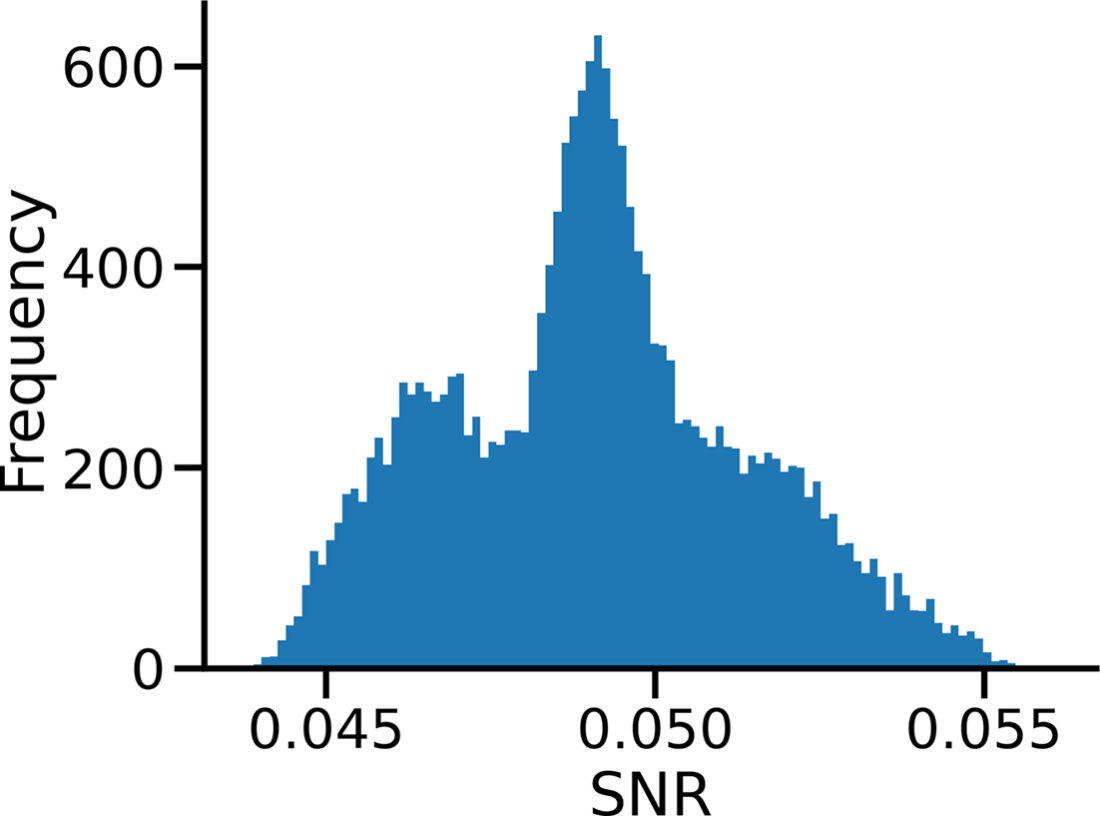
Distribution of SNR for synthetic data of TRPV1. Each SNR is calculated from equation (37) before the expectation over (here 20 000) images. Each simulated measurement contains per pixel i.i.d. Gaussian white noise (σ = 3).

The discussion section in Kimanius *et al.* (2024 ▸) anticipates and calls for more discussion around validation, because the current pipeline of visually comparing scalar density maps against features from an expected underlying atomic model(s) is a fruitful heuristic that is not to be underestimated. Schwab *et al.* (2024 ▸) learned a per-image 3D deformations of a Gaussian pseudo-atoms model and noted that an atomic model prior introduced unacceptable bias, but did include coordinate-based regularizers enforcing smoothness of deformations, local isometry and repulsion. They also estimate the error of deformations by training two separate deformation decoders and checking for agreement (one training subset is a validation subset for the other network). We also validated with two networks in Fig. 11, and caution that the inductive bias that we observed in early training would confound self-consistency estimates at face value in pathological circumstances; however, in our case the inflated FSC value dissipated after 50–100 particles and did not seem to be an issue otherwise.

On the inverse model/inference side, we hope to see more empirical comparisons with state-of-the-art architectures (and objectives) such as skip connections, transformers, diffusion models, geometric equi/invariances and topological neural networks (Bronstein *et al.*, 2021 ▸; Papillon *et al.*, 2023 ▸), although we note that in the case of Schwab *et al.* (2024 ▸) various architectures (with residual connections, more linear layers or 2D convolutions) or optimizing a local per-image encoding (*i.e.* non-amortized inference) were reported to yield similar results. Furthermore, cryo-electron tomography (cryo-ET) or *in situ* cryo-EM, where electron micrographs of slices of cells are the imaging target rather than purified biomolecules, has open problems at which we can point the end-to-end framework we have employed here in *InstaMap*. Indeed, instant-NGP has already been applied to cryo-ET data (Wang *et al.*, 2023 ▸).

As mentioned in Section 1, the concurrent work *CryoNeRF* (Qu *et al.*, 2025 ▸) is an extremely similar approach, with minor differences in implementation, which we deliniate in Section A8. We are intrigued by these differences and interested to explore the many permutations of network architecture, instant-NGP hyperparameters, learned image embeddings, and interpretable geometric transformations on coordinates in future work. Cryo-EM is a challenging but meaningful scientific inference problem, and we look forward to its synergy with methodologies that have proved fruitful in computational research.

## Figures and Tables

**Figure 12:**
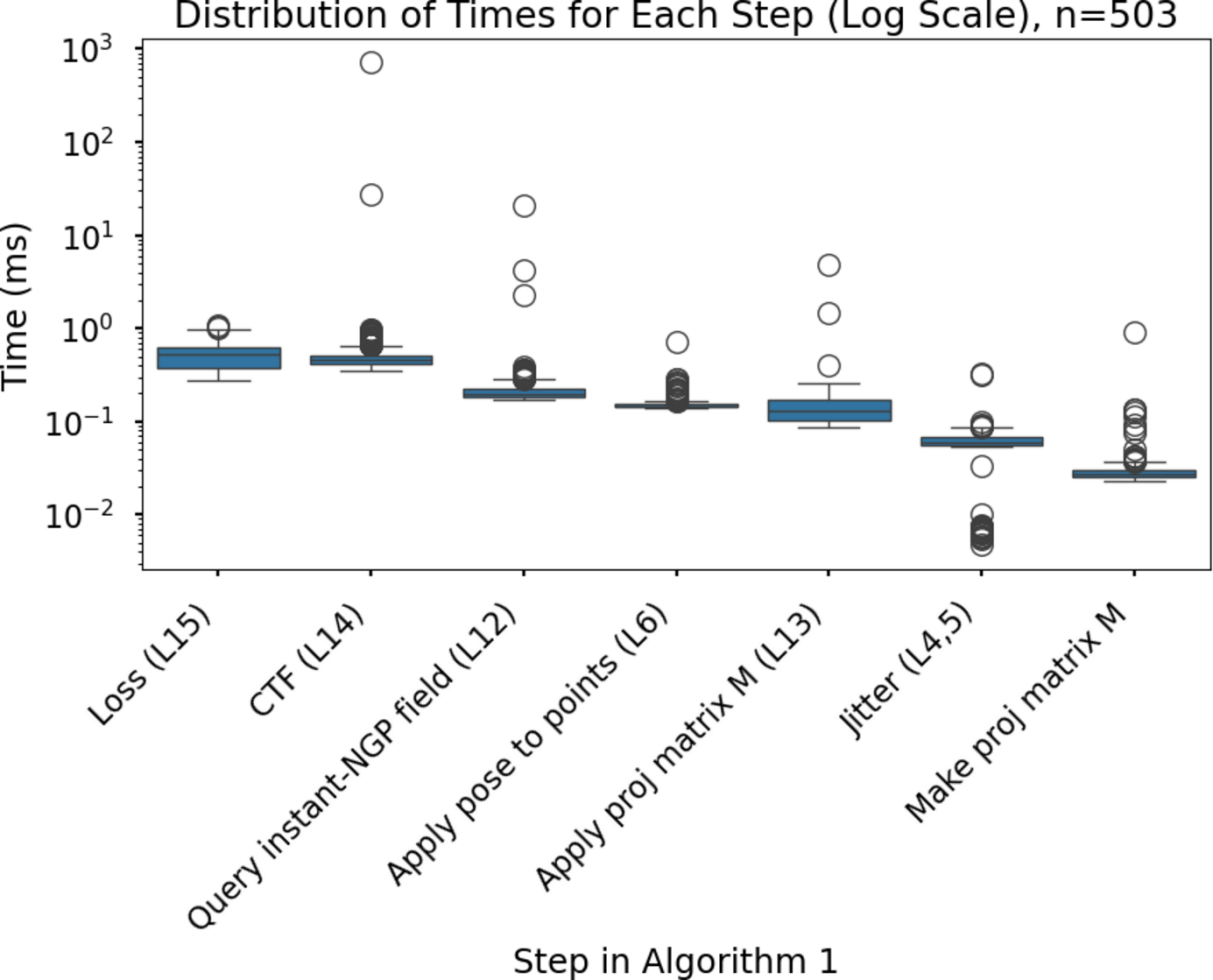
Run-time bottlenecks. Various steps in Algorithm 1 are shown with their corresponding line numbers, ranked from longest to shortest time.

**Figure 14:**
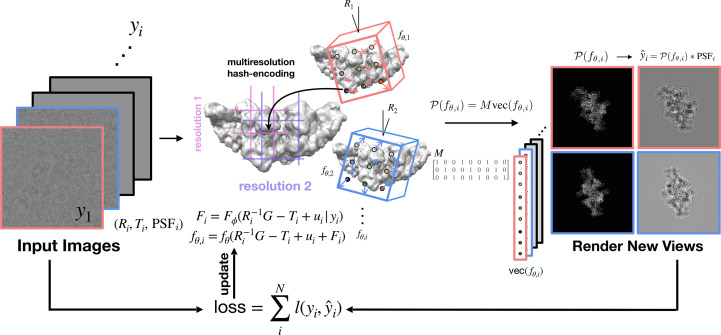
*InstaMap* models heterogeneity by bending space. Cryo-EM images *y_i_* (left) with annotated pose and imaging parameters (*R*_*i*_, *T*_*i*_, PSF_*i*_) are used for gradient-based learning. A vector field in a fixed frame is queried at the rotated, shifted and jittered grid to provide a per-image SE(3) equivariant output *F*_*i*_. Space is bent via an additive perturbation on the corresponding rotated, shifted and jittered grid. The remaining pipeline is per Fig. 1 ▸.

**Table 1:** Number of trainable parameters versus hash-map size The number of trainable parameters arises from the number of levels (*L*) and also includes those from the decoder (two-layer MLP with 128 neurons each) and assumes an output volume size of *n*^3^ = 160^3^.

No. of trainable parameters	Hash-map size
35000	2^10^
75000	2^12^
200000	2^14^
610000	2^16^
1800000	2^18^
5100000	2^20^
11400000	2^22^

**Table 2:** Heterogeneity confusion matrices The number of images in the predicted class and true class are shown, with corresponding accuracy, sensitivity/recall, specificity and precision for (vector field; image pixels): (98.72%; 49.97%), (98.83%; 99.85%) and (98.62%; 0.10%), (98.63%; 49.99%), respectively.

	Predicted vector-field cosine similarity		Observed image pixels *L*_2_	
	Predicted		Predicted	
Actual	+	−	+	−
+	1937	23	1957	3
−	27	1933	1958	2

## Data Availability

The code is available at https://github.com/flatironinstitute/InstaMap.

## Footnotes

1 *f*_TW_, *f*_SH_, *f*_θ_, *f*_OB_ and *f*_ω_ use the same tinycudann PyTorch binding: see the documentation.

2 We did use *l*_MSE_ for some heterogeneity experiments.

3 See *Methods*, *Convection operator*: ‘the flow in 3*DFlex*, **u**_*i*_, is a forward mapping from canonical coordinates in *V* to the deformed coordinates in *W*_*i*_’.

## Appendix A

### A1. Computation

#### A1.1. Instant-NGP configuration

We used the following configuration settings: otype=Grid, type=Hash, n_levels=8 (the size of the coarsest grid), log2_hashmap_size=17 (the number of entries in the hash table at each level), base_resolution=8, per_level_scale=exp[log(size/base_size)/n_levels − 1] and size=160 (TRPV1), size=288 (apoferritin) or size=256 (thyroglobulin). Following the original paper (Müller *et al.*, 2022 ▸), we used a two-layer MLP decoder with 64 (or 128) neurons and ReLU activations. The departures from this instant-NGP architecture are mentioned in the text.

#### A1.2. Heterogeneity encoder architecture

We parametrized the per-image heterogeneity encoder via a MLP neural network, taking the flattened image as input and outputting a vector field on the regular grid of shape (3, *n*_*F*_, *n*_*F*_, *n*_*F*_), with *n*_*F*_ = 64, corresponding to a downsampling factor of 8 for the 256 box-sized images. The MLP had three layers of 32 neurons, with ReLU activations, which corresponded to 2.14 million trainable parameters.

#### A1.3. Optimization

We used the AdamW optimizer, torch.optim.AdamW, with a learning rate of 0.001 times the batch size (1 or 2 in our studies due to GPU memory limitations) and zero weight decay. We used a linear warm-up schedule, torch.optim.lr_scheduler, with a scheduler decay defined by torch.optim.lr_scheduler.StepLR(..., gamma=0.7).

#### A1.4. Codebase

The code is available at https://github.com/flatironinstitute/InstaMap. We wrote our code base in PyTorch. We made use the deep-learning framework PyTorch Lightning and the hierarchical configuration systems OmegaConf (https://omegaconf.readthedocs.io) and Hydra (https://hydra.cc/).

#### A1.5. Compute

All experiments were performed on a high-performance computer cluster with 200 GB of CPU RAM and a single NVIDIA GPU (a V100, A100 or H100, depending on availability). Training time lasted from minutes to several hours, depending on the experiment.

#### A1.6. Training speed

For homogeneous training speeds, see Table 1 ▸ and Figs. 5 ▸ and 11 ▸. With the heterogeneity network *F*_φ_ the training speed decreased to about ∼10–12 gradient steps per second, with one image per gradient step.

A run-time breakdown of various steps from Algorithm 1 is shown in Fig. 12 ▸, showing that computing the code blocks that (i) compute the loss, (ii) generate and apply the CTF and (iii) query instant-NGP are the largest three bottlenecks (in decreasing order), while making the projection matrix, which is performed in each gradient step for the points *G*, which may change due to masking, is even slower than jittering the points, which involves vectorized sampling of uniform noise and one vectorized addition.

### A2. CTF

The CTF has the form

where

where *k* = (*k*_*x*_, *k*_*y*_) are the reciprocal coordinates, δ_1_, δ_2_, θ_δ_ are the defoci and their astigmatism angle, θ_ps_ is the phase shift, λ is the electron wavelength, *C*_*s*_ is the spherical aberration and the spherical aberration-corrected defocus Δδ = ½{δ_1_ + δ_2_ + (δ_1_ − δ_2_)cos[2(θ_*k*_ − θ_δ_)]}, θ_*k*_ = atan2(*k*_*x*_, *k*_*y*_).

This convolution subpart of the forward model of image formation, , is differentiable, and thus automatic differentiation propagates the gradient from the loss function to θ.

### A3. FSC

The Fourier shell correlation (van Heel & Harauz, 1986 ▸)is defined between two 3D objects and maps each discrete bin in Fourier basis to a real-valued correlation, .

where *F*_(·)_(*k*) is the 3D Fourier transform of the 3D object at frequency shell , (·)^†^ denotes the conjugate transpose and Re is the real-spaced projection operator.

### A4. Loss functions

The loss function *l*_CC_ is equivalent to *l*_MSE_, when the two norm terms of *y* and  are neglected, and when *w* is the identity: *w*[(·)] = (·).

The  cross term is made invariant to a global multiplicative and additive scalar by applying, to both *y* and , the function *w* which subtracts the mean and divides by the standard deviation,

The BioEM loss was originally derived with a flat prior according to the equation

where

Completing the square again to perform the integral over *N*, we see that equation (19) is equivalent to equations (4)–(9) in the Supplementary Information of Cossio & Hummer (2013 ▸).

where

Equation (26) is not invariant to global multiplicative scaling of . While the −*b*_2_/4*a*_2_ + *c*_2_ term is invariant, the term , such that , which is maximized in the limit . This causes numerical instabilities in an end-to-end framework, since optimizing *f*_θ_ can drive . Indeed, we observed this in numerical experiments: with the TRPV1 empirical data set, after about a few thousand gradient steps the output of *f*_θ_ was sufficiently close to zero that it rounded to all zeros in finite precision. This caused numerical instabilities that were insurmountable by injecting random noise.

The more general loss function that applies the saddle-point approximation is likewise not invariant to global multiplicative scaling, and  gives .

This motivated us to derive a related loss, but with a prior that was centered around a value that was numerically stable. The integral can be performed analytically for a Gaussian prior, which changes the integral over *N* in equation (25):

where

### A5. Data sets

#### A5.1. Homogeneous reconstruction of TRPV1 and apoferritin

Synthetic data were rendered for the ion channel TRPV1 from an atomic model from PDB entry 3j5p. We approximated the density with a mixture of Gaussians placed at each atom coordinate, corresponding to the parametrization in Lobato & Van Dyck (2014 ▸), and added Gaussian white noise at fixed σ = 3. Experiments in this paper are at a signal-to-noise ratio (SNR) of ∼0.045 ± 0.002; see Fig. 13 ▸ and equation (10 ▸) for more details. The pixel size (1.2 Å), box size (160), poses and microscope parameters were chosen to match the empirical data set we used (EMPIAR-10005), which was re-picked in-house. For our second specimen, we chose apoferritin due to its ability to reach high resolution and its ubiquity as a reference sample. All experiments in this paper for apoferritin are with empirical data (EMPIAR-10421) and use the published pixel size (0.816 Å) and box size (288). We split the synthetic and empirical data sets for training and validation in the ratio 95:5.

#### A5.2. Heterogeneous reconstruction of thyroglobulin

Synthetic data were rendered from a course-grained model of thyrogobulin from Astore *et al.* (2023 ▸). States were created by linear extrapolation between the coordinates of two extrema (; *n*_*a*_ is the number of coarse-grained pseudo-atoms) of the states employed by Astore *et al.* (2023 ▸), as follows: one state (A) was an extremum (*r*_*A*_ = *r*_1_) and the other state (B) was generated by linearly extrapolating the coordinates along the motion defined by the difference between the two extrema (Δ*r* = *r*_2_ − *r*_1_), using a scaling factor of 4, such that *r*_*B*_ = *r*_*A*_ + 4Δ*r*. We approximated the density with a mixture of Gaussians placed at each atom coordinate, corresponding to the parametrization in Lobato & Van Dyck (2014 ▸), and added Gaussian white noise at fixed σ = 0.1 (more signal than in other experiments). The poses and microscope parameters are taken from the empirical distribution of the data set EMPIAR-10005 (TRPV1). We used a pixel size of 1.5 Å and a box size of 256.

### A6. SNR of synthetic data

We computed the SNR using equation (37). Strictly speaking, each simulated image has its own SNR, because the variance in the signal is pose-dependent. We show a representative distribution of SNRs in Fig. 13 ▸.

### A7. Heterogeneity

Heterogeneity is inferred through amortized inference, such that each image *y*_*i*_ has a corresponding SE(3) equivariant perturbation vector field, *F*_*i*_, which renders into a 3D volume *f*_θ,*i*_(*G*_*i*_ + *F*_*i*_) in a reference frame corresponding to *R*_*i*_ = *I*, *T*_*i*_ = 0 (Fig. 14 ▸). Fig. 10 ▸ shows vector-field similarities for 1960 images of states A and B (each), illustrating the accuracy for different poses, including those where the pose obscures the shape. We compare per-image vector fields through cosine similarity, which has a maximum of 1 and minimum of −1. Based on these similarities, we define a per-image summary statistic that measures accuracy:

The predicted vector fields were inferred from a test set of images. The departure of the cosine similarity matrix, , from a block diagonal matrix (all 1s on the two diagonal blocks of 1960 × 1960; a lower value on the off-diagonal blocks), illustrates the generalization of amortization. Furthermore, in order to demonstrate how the heterogeneity could be analyzed without access to the ground truth we performed clustering (sklearn.cluster.SpectralClustering(n_clusters=2, affinity=‘precomputed’, assign_labels=‘kmeans’).fit_predict(...)} on the cosine similarities of the vector fields (which requires no access to ground truth), and used these predictions to condition on in Fig. 9 ▸. We verified that the false positives (23/1960 = 1.17%) and false negatives (27/1960 = 1.38%) were low, and show the confusion matrix in Table 2 ▸. The cosine similarity between two elements of vector fields is a suitable inner product on this space, and efficient to compute (seconds for thousands of vector fields). We compute a similarity matrix on the image pixels themselves (*L*2 norm of the residual), taking no care to perform in-plane rotation, and perform out-of-the-box spectral clustering as outlined above. Table 2 ▸ quantifies the poor performance of classifying based on an image *L*2 similarity, thereby underlining the fittingness of an SE(3) invariant vector field as a per-image latent embedding. Furthermore, the vector field itself could be analyzed in more detail: through masking around a region of interest, or dimensionality-reduction techniques such as principal component analysis or uniform manifold approximation and projection (UMAP), as is commonly performed for neural network encodings of heterogeneity.

### A8. Comparison with concurrent work employing instant-NGP

The projection in Qu *et al.* (2025 ▸) is performed with a torch.scatter operation along a ray of density values in the imaging direction, rather than multiplication with a projection matrix (type torch.sparse_csr_tensor) to the same effect. They model heterogeneity through concatenating the Grid encoding (of coordinates) with an encoding of each image (from a ResNet architecture), before decoding that with a light-weight MLP. Thus, rather than employing an explicit geometric transformation on coordinates, and querying into a learned reference volume, they map 2D image and 3D coordinates to 3D scalar values. Their instant-NGP configuration (n_levels: 16, n_features_per_level: 2, log2_hashmap_size: 19, base_resolution: 16, per_level_scale: 1.4472692012786865) is similar to ours (n_levels: 8, log2_hashmap_size: 19–22, base_resolution: 8, per_level_scale: exp[log(size/base_resolution)/n_levels −1]) except that we use a smaller n_levels, an equal or larger log2_hashmap_size, a smaller base_resolution and a smaller per_level_scale [1.204 for TRPV1 (size 160), 1.240 for thyroglobulin (size 256) and 1.249 for apoferritin (size 288)]. The full meaning of these parameters is explained in the instant-NGP documentation (https://github.com/NVlabs/tiny-cuda-nn/blob/master/DOCUMENTATION.md#grid).
